# Comparative Ca^2+^ channel contributions to intracellular Ca^2+^ levels in the circadian clock

**DOI:** 10.1016/j.bpr.2021.100005

**Published:** 2021-07-21

**Authors:** Amber E. Plante, Vishnu P. Rao, Megan A. Rizzo, Andrea L. Meredith

**Affiliations:** 1 Department of Physiology, University of Maryland School of Medicine, Baltimore, Maryland

## Abstract

Circadian rhythms in mammals are coordinated by the central clock in the brain, located in the suprachiasmatic nucleus (SCN). Multiple molecular and cellular signals display a circadian variation within SCN neurons, including intracellular Ca^2+^, but the mechanisms are not definitively established. SCN cytosolic Ca^2+^ levels exhibit a peak during the day, when both action potential firing and Ca^2+^ channel activity are increased, and are decreased at night, correlating with a reduction in firing rate. In this study, we employ a single-color fluorescence anisotropy reporter (FLARE), Venus FLARE-Cameleon, and polarization inverted selective-plane illumination microscopy to measure rhythmic changes in cytosolic Ca^2+^ in SCN neurons. Using this technique, the Ca^2+^ channel subtypes contributing to intracellular Ca^2+^ at the peak and trough of the circadian cycle were assessed using a pharmacological approach with Ca^2+^ channel inhibitors. Peak (218 ± 16 nM) and trough (172 ± 13 nM) Ca^2+^ levels were quantified, indicating a 1.3-fold circadian variance in Ca^2+^ concentration. Inhibition of ryanodine-receptor-mediated Ca^2+^ release produced a larger relative decrease in cytosolic Ca^2+^ at both time points compared to voltage-gated Ca^2+^channels. These results support the hypothesis that circadian Ca^2+^ rhythms in SCN neurons are predominantly driven by intracellular Ca^2+^ channels, although not exclusively so. The study provides a foundation for future experiments to probe Ca^2+^ signaling in a dynamic biological context using FLAREs.

## INTRODUCTION

Ca^2+^ signaling is essential for the production of time-keeping signals in the mammalian circadian clock, which is located in the suprachiasmatic nucleus (SCN) of the hypothalamus. Ca^2+^ is required for SCN neurons to maintain 24-h rhythms in clock gene expression (1,2) and action potential activity (3–6). These main SCN output signals ultimately control the timing of downstream physiological processes (7–9) and behaviors (10–12). Ca^2+^ signaling is also required for SCN neurons to respond to external inputs (13–20) that lead to shifts in molecular (21), cellular (14,22), and behavioral rhythms (23).

A circadian pattern in intracellular free Ca^2+^ has been identified in both SCN neurons and glia (24–29). SCN neurons exhibit a circadian rise in cytosolic Ca^2+^ concentration that typically reaches a peak during the day, ~0–5 h before the peak in action potential firing, and is reduced at night (24,27,30–32). These rhythmic Ca^2+^ signals can be observed in single SCN neurons as well as from whole SCN slices (19,24,28,30,32–35). However, the major Ca^2+^ channels that drive these Ca^2+^ rhythms are still under investigation.

Previous studies have implicated multiple Ca^2+^ channel subtypes that contribute to Ca^2+^ signaling in SCN neurons (27). Membrane depolarization stimulates Ca^2+^ influx by activating voltage-gated Ca^2+^ channels (VGCCs) including L-, N-, P/Q-, R-, and T-type channels (2–5). Ca^2+^ release from intracellular stores in the endoplasmic reticulum (ER) is mediated by ryanodine receptors (RyR2 and RyR3) (24,36–38). IP_3_ signaling stimulates Ca^2+^ release from the ER by activating inositol 1,4,5-trisphosphate receptors (IP_3_Rs) (2,39). Prior studies have shown that cytosolic Ca^2+^ levels may be mediated in part by Ca^2+^ release from intracellular ER stores, as pharmacological inhibition of RyRs produces a large decrease in cytoplasmic Ca^2+^ (24,40,41). However, Ca^2+^ influx through the plasma membrane from voltage-gated Ca^2+^ channels may also contribute. Inhibition of L-type voltage-gated Ca^2+^ channels with nimodipine or action potential firing with the Na^+^ channel blocker tetrodotoxin partially reduce cytoplasmic Ca^2+^ (40). To date, no single study has directly compared the contributions of the main plasma membrane and intracellular Ca^2+^ channels at both the peak and trough of the circadian cycle from intact SCN slices.

This study utilizes a newly developed fluorescent biosensor to provide a quantifiable and direct comparison for the contributions of voltage-gated and intracellular Ca^2+^ channels to daytime (peak) and nighttime (trough) Ca^2+^ levels in SCN neurons from intact brain slice cultures. Polarization inverted selective-plane illumination microscopy (piSPIM) was used to measure Ca^2+^ concentration within SCN using a ratiometric, neuronally expressed Ca^2+^ sensor, Venus FLARE-Cameleon (Venus-cp172Venus FLARE-Cameleon) (42). The Venus FLARE-Cameleon sensor is a fluorescence resonance energy transfer (FRET)-based fluorescence anisotropy reporter (FLARE) (42). Ca^2+^ concentrations were estimated from in situ calibration of Venus FLARE-Cameleon fluorescence anisotropy signals in SCN slices. Pharmacological inhibitors targeting the major Ca^2+^ channel subtypes were applied during the peak and trough phases of the diurnal cycle to evaluate the impact of different Ca^2+^ sources on Ca^2+^ levels. These data revealed a peak-to-trough difference in cytosolic Ca^2+^ concentration that was higher during the day, with ryanodine receptors providing the largest contribution at both times of the diurnal cycle.

## MATERIALS AND METHODS

### Animals and ethical approval

Wild-type C57BL/6J mice were bred in a standard 12:12-h light-dark cycle. Male and female mice were killed for experiments via decapitation at postnatal day 4. All procedures involving mice were conducted in accordance with the University of Maryland School of Medicine Animal Care and Use Guidelines and approved by the Institutional Animal Care and Use Committee.

### Organotypic slice culture and viral transduction

Brains were dissected during the light cycle as described previously (3). Coronal sections of the hypothalamus (300 *μ*m) were made on a manual tissue chopper (Stoelting, Wood Dale, IL) in ice-cold dissection medium containing bicarbonate-free Dulbecco’s modified Eagle’s medium (12100-046; Gibco, Gaithersburg, MD), 10 mM HEPES (pH 7.3), 100 U/mL penicillin/streptomycin (30-002-CI; MediaTech, Manassas, VA), and 2 mM L-glutamine (25-005-CI; MediaTech). Slices containing the SCN (one per animal) were cultured as organotypic interface explants (43).

For piSPIM experiments, SCN slices were plated onto filter membranes (PICM0RG50; Millipore Sigma, Burlington, MA) in 35-mm culture dishes (353001; Corning, Corning, NY) with 1.2 mL culture medium containing minimal essential medium (11095-080; Gibco), 25 mM HEPES (pH 7.3), 25% horse serum (16050-130; Gibco), 28 mM D-glucose (G8270;Millipore Sigma), 10 U/mL penicillin/streptomycin (30-002-CI; MediaTech), and 2 mM L-glutamine (25-005-CI; MediaTech). Cytosine *β*-D-arabinofuranoside (Ara-C, 20 *μ*M; C6645; Millipore Sigma) was added to culture medium starting on culture day 2 to inhibit glial cell growth. Immediately after plating, slices were transduced with 1 *μ*L of adeno-associated viral vector (AAV, serotype 1) containing Venus-cp172Venus FLARE-Cameleon biosensor DNA (42) (AAV1.hSyn1.Vencp172Ven Cameleon; stock 2.18 × 10^12^ vg/mL; plasmid #pOTTC1612; Genetic Engineering and Viral Vector Core, National Institute on Drug Abuse, Baltimore, MD). Neuron-specific expression was driven by the human synapsin 1 (hSyn1) promoter (44). Slices were maintained for 14–21 days in a humidified incubator at 37°C (5% CO_2_) with 100% of the culture medium exchanged every ~72 h.

A subset of slices transduced with Venus FLARE-Cameleon AAVs were plated onto multielectrode arrays on culture day 10 as described previously (43,45). SCN slices cultured on filters were excised from the surrounding filter and flipped (SCN side down) onto multielectrode arrays pretreated overnight with 500 *μ*L of 0.1 mg/mL collagen (C8919; Sigma-Aldrich, St. Louis, MO) and maintained in culture medium as described in Supporting materials and methods.

### piSPIM imaging of fluorescence anisotropy

Imaging experiments were conducted in 6-h time windows using the peak and trough of firing rhythms as the reference point, in which images were obtained between 5 h before to 1 h after the time of the peak or the trough in action potential firing. Filter sections with SCN slices were excised, rinsed in phosphate-buffered saline, transferred to the microscope chamber, and equilibrated for 20–30 min in 6 mL of prewarmed imaging solution containing 125 mM NaCl, 8 mM NaOH, 5 mM KCl, 1 mM MgCl_2_, 20 mM HEPES, 5 mM D-glucose, and 2.5 mM CaCl_2_ (pH 7.20 ± 0.01 at 35°C). Fluorescence anisotropy imaging was performed on a polarization inverted selective-plane illumination microscopy (piSPIM) microscope with stage-scanning capability assembled and aligned as described previously (46–48). The collection arms of the microscope were fitted with filter wheels containing emission filters and an image splitting device, OptoSplit II (Cairn, Faversham, UK), to separate parallel (*P*) and perpendicular (*S*) polarizations. The microscope was housed in an environmentally controlled incubator (Okolab, Ambridge, PA) maintained at 37°C. Automated stage and piezo focus control hardware elements were purchased from Applied Scientific Instruments (Eugene, OR). Camera and piezo electronics were controlled using Micromanager software (available at https://micro-manager.org/) (49) on a Z840 workstation (Hewlett Packard, Palo Alta, CA). Volumetric images (16-bit grayscale) were collected on a Nikon Eclipse TE2000-U microscope with water-dipping objectives (MRD07420, 40×, 0.8 NA; Nikon, Tokyo, Japan) and a digital camera (Flashv4 Orca, C13440; Hamamatsu, Hamamatsu, Japan) as stack files with 20 image slices per volume (1-*μ*m spacing, 512 × 1024 pixels per image slice, 332-nm pixel width and height, 2 × 2 binning). Samples were excited in 10-s (KCl experiments) or 30-s (Ca^2+^ inhibitor experiments) intervals with a 488-nm laser. Images were collected from a ~170 × 340 × 20 *μ*m area within the center of the SCN, which was visually identified under brightfield illumination at 4× magnification using the optic chiasm and third ventricle as reference landmarks. After baseline control images were acquired, imaging solution (100–200 *μ*L) was removed from the bath, mixed with the appropriate amount of drug stocks or dimethyl sulfoxide (DMSO) (<0.01%, D2650; Sigma-Aldrich), and reapplied to the bath chamber. The temperature of the bath solution was 35 ± 0.1°C.

### piSPIM image processing and data analysis

Images were processed and analyzed according to Ross et al. (42,50) with some modifications using ImageJ (FIJI) macros and script executed in Python (v3.7). Volumetric image stacks were split to separate *P* and *S* channels. Corresponding *P* and *S* image stacks (512 × 1024 pixels) were aligned using a Python script and separated into individual images. The median grayscale value of the background intensities for each image was calculated and subtracted. An adaptive local thresholding method was used to obtain a binary clipping mask to separate cell signals from image background. The local threshold value for each pixel was calculated using the Gaussian-weighted sum of the neighborhood pixel intensities (51,52). Anisotropies (*r*) were calculated using pixel intensities above the threshold value from the corresponding background-subtracted *P* and *S* images using the equation (53)

$$r=\frac{P-gS}{P+2gS}.$$

The g-factor constant (*g*) was measured using an isotropic fluorescein solution and calculated to account for the difference between *P* and *S* channel transmission efficiencies as previously described (50). The *r*-values for each image were summed across all images in each stack and plotted as a histogram distribution. A single mean *r*-value for each image stack was calculated with a Gaussian fit of the *r* histogram distribution in Prism v8.4 (GraphPad Software, San Diego, CA). Scripts for automated image alignment, background subtraction, pixel thresholding, and *r*-value calculations were executed in Python.

### In situ calcium calibration

Ca^2+^ buffering solutions were prepared using the method described in McGuigan et al. (54). To ensure EGTA concentrations in Ca^2+^-EGTA and EGTA solutions were identical, a 2× EGTA stock solution containing all ingredients (except for NaOH and CaCl_2_) was prepared and split into two volumes. CaCl_2_ and NaOH were added to one volume and diluted to obtain a 1× Ca^2+^-EGTA solution containing 125 mM NaCl, 44 mM NaOH, 5 mM KCl, 2 mM KOH, 1 mM MgCl_2_, 20 mM HEPES, 1.8 mM 2-deoxy-D-glucose, 5 mM EGTA, 5 mM CaCl_2_, 0.01 mM rotenone, 0.01 mM ionomycin, and 0.01 mM cyclopiazonic acid (CPA) (pH 7.20 ± 0.01 at 35°C). NaOH and HCl were added to the second volume to produce a final 1× EGTA (zero free Ca^2+^) solution containing 125 mM NaCl, 44 mM NaOH, 5 mM KCl, 2 mM KOH, 1 mM MgCl_2_, 20 mM HEPES, 1.8 mM 2-deoxy-D-glucose, 5 mM EGTA, 0.01 mM rotenone, 0.01 mM ionomycin, and 0.01 mM CPA (pH 7.20 ± 0.01 at 35°C). The appropriate quantities of Ca^2+^-EGTA and EGTA solutions were mixed to obtain solutions with known free Ca^2+^ concentrations calculated with WebMaxC standard (available online at https://somapp.ucdmc.ucdavis.edu/pharmacology/bers/maxchelator/webmaxc/webmaxcS.htm). SCN slices were equilibrated in Ca^2+^ buffer solutions at least 20 min before imaging. All imaging solutions were prepared with Ca^2+^-free liquid-chromatography mass-spectrometry (LC-MS)-grade water (WX0001-6; Sigma-Aldrich). The dissociation constant (*K_d_*) and Hill coefficient (*n*) were determined by fitting a plot of the *r* vs. Ca^2+^ concentration data in Prism (GraphPad Software) with the equation

$$r=rmin+\frac{rmax-rmin}{1+{\left(\frac{K_{d}}{\left[{\text{Ca}}^{2+}\right]}\right)}^{n}}.$$

### Statistics

Statistical tests were performed in Prism v8.4 (Graphpad Software). Changes in anisotropy values across baseline time points were tested with a two-way analysis of variance (ANOVA) with repeated measures. Student’s *t*-tests (two tailed) were used to determine significant differences in anisotropy values and Ca^2+^ concentrations between peak and trough time points. One-way ANOVA with Bonferroni’s post hoc tests were used to determine significant differences in ΔCa^2+^ between conditions at each time point. Paired *t*-tests (two tailed) were used to test for changes in anisotropy and Ca^2+^ concentration between baseline and drug conditions for individual SCN slices at each condition. Significant differences in GCaMP6f fluorescence across multiple peak and trough time points were tested with a two-way, repeated-measures ANOVA and Bonferroni’s post hoc test using the F/Fmax-values from individual cells for all slices across time points.

### Pharmacology

Pharmacological reagents were used at final concentrations of 10 *μ*M nimodipine (Nim; N150; Alomone Labs, Jerusalem, Israel), 10 *μ*M dantrolene (Dan; D9175; Sigma-Aldrich), 30 *μ*M CPA (C-750; Alomone Labs), 3 *μ*M *ω*-conotoxin GVIA (ConoGVIA; C-300; Alomone Labs), 200 nM *ω*-agatoxin IVA (AgaIVA; STA-500; Alomone Labs), 30 *μ*M NiCl_2_ (Ni^2+^; 22387; Sigma-Aldrich), 10 *μ*M ionomycin (407951; Sigma-Aldrich), 10 *μ*M rotenone (R8875; Sigma-Aldrich), and 50 mM KCl (P9333; Sigma-Aldrich). Reagent stocks (1000×) were prepared in DMSO (Nim, Dan, CPA, ionomycin, rotenone) or water (ConoGVIA, AgaIVA, Ni^2+^) and stored at −20°C. KCl was prepared as a 4 M HEPES-buffered stock solution.

## RESULTS

In the ex vivo organotypic slice preparation, the isolated SCN exhibits intrinsic circadian rhythmicity. First, rhythms in long-term spontaneous action potential activity were recorded by multielectrode array (Supporting materials and methods) to verify robust intrinsic circadian rhythms in cultured SCN slices before imaging. After establishing the diurnal phase using action potential firing, standard confocal imaging was used to verify intracellular Ca^2+^ was also rhythmic under these experimental conditions using the Ca^2+^ sensor GCaMP6f (Fig. S1). These data were then used to determine the time windows for quantitative Ca^2+^ imaging using piSPIM.

**FIGURE 1:**
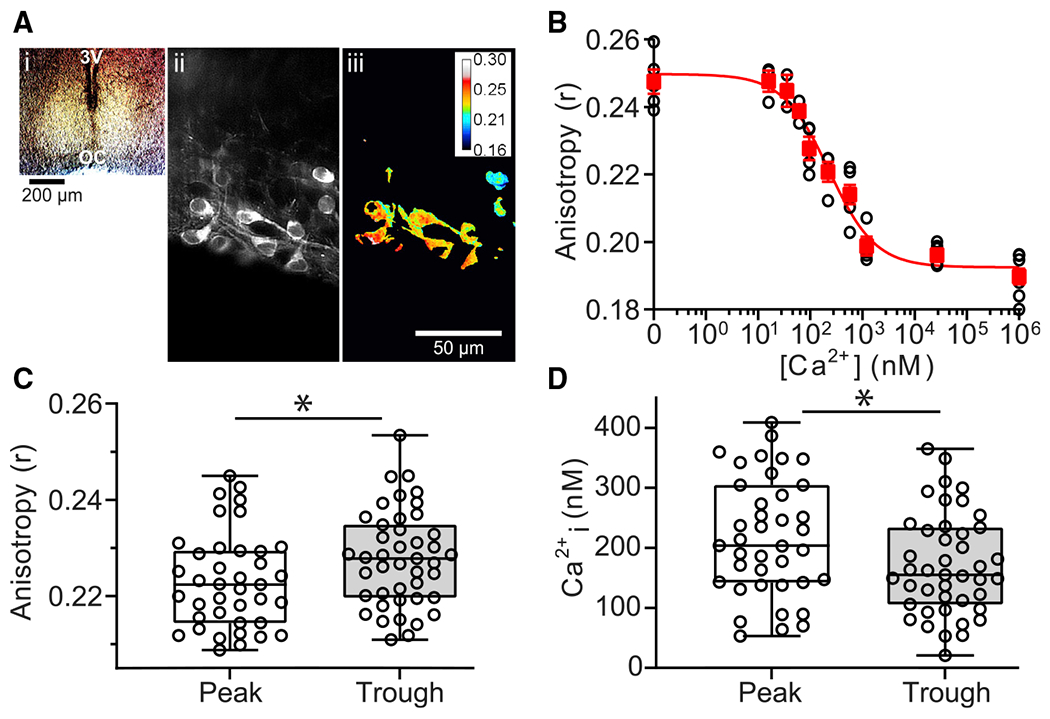
Neuronal Ca^2+^ concentrations at peak and trough in SCN. (*A i*) Brightfield image of SCN at 4× magnification. (*A ii*) piSPIM image of polarized fluorescence signals from SCN neurons expressing Venus FLARE-Cameleon at 40× magnification. (*A iii*) Pseudocolor map of anisotropy values from the image in (*A ii*) calculated from parallel and perpendicular polarized fluorescence signals after image thresholding and background subtraction. (*B*) In situ calibration of the Venus FLARE-Cameleon sensor in SCN neurons. Anisotropy values from regions of SCN slices incubated in solutions with known free Ca^2+^ (0–1 mM) are plotted against Ca^2+^ concentration. Data points (*open black circles*) representing anisotropy measurements from separate imaging regions were fitted with a nonlinear regression (*red line*) overlayed with the mean ± SEM of the anisotropy values from all slices in each Ca^2+^ condition (*closed red squares*). N = 2–4 slices per Ca^2+^ condition with one to three imaging regions per slice. (*C*) Plots of median, 25th and 75th percentile (*boxes*), and minimal and maximal (*whiskers*) anisotropy values at peak and trough time points. (*D*) Box plots of estimated peak and trough Ca^2+^ concentrations calculated from anisotropy values in (*C*). Compared to the trough, peak anisotropy was significantly decreased (*p* = 0.04) and peak Ca^2+^ concentration was significantly increased (*p* = 0.02). *p < 0.05, unpaired Student’s *t*-test. Data points represent measurements from individual SCN slices (one imaging region per slice). N = 39 slices for peak, N = 43 slices for trough.

To measure intracellular Ca^2+^ using Venus FLARE-Cameleon (42), SCN slices were cultured on filter membranes (Fig. 1 A i) and transduced with AAVs containing Venus FLARE-Cameleon cDNAs (42) expressed under the neuron-specific hSyn1 promoter (44). Ca^2+^ binding to the Venus FLARE-Cameleon protein induces FRET between the two Venus fluorophores (55–59), which is detected as a decrease in the polarization (anisotropy) of emitted light from the sensor (42,53,59–61). FRET-based measurements from Venus FLARE-Cameleon provide a ratiometric quantification of Ca^2+^ concentration that is insensitive to variation in expression levels, cell morphology, illumination, or experimental preparations. Thus, this biosensor circumvents the variability in measurements that are based on fluorescence intensity (42,53,55,56,59,62,63,64), enabling quantitative measurements of Ca^2+^ that are comparable across experimental time points and different SCN slices.

Fluorescent signals from neurons expressing the Venus FLARE-Cameleon biosensor were clearly detectable within the SCN (Fig. 1 A ii). Volumetric images of the polarized fluorescence signals were collected from a cubic area of the SCN (Fig. S2). A local threshold, calculated based on the sum from a Gaussian window, was applied to each image to delineate cell signals (above threshold) from background (below threshold). Anisotropies were calculated from integrating the signal from all pixels above the threshold in each image, which were summed across all images (20 images per stack) to provide a single anisotropy distribution per image stack (Fig. 1 A iii). Anisotropy histograms were fitted with a Gaussian distribution to calculate a single mean anisotropy value encompassing signals from all cells within the imaging region.

Because the relationship between anisotropy values and Ca^2+^ concentration can be sensitive to variations in temperature and pH (65), the Ca^2+^ concentration and fluorescence anisotropy for Venus FLARE-Cameleon was calibrated in situ from SCN slices incubated in buffered standards of known Ca^2+^ concentration at 35°C (Fig. 1 B). For in situ calibration experiments, ionomycin, which permeabilizes the cell membrane to Ca^2+^; rotenone, an ATP inhibitor; and CPA, a SERCA-ATPase inhibitor, were added to the bath solution to limit homeostatic compensation and promote the clamping of intracellular Ca^2+^ concentration (66). We found that the dissociation constant (K_d_ = 230 nM) and hillslope (n = −1.0) values obtained from this in situ Ca^2+^ calibration curve were similar to those previously reported for this sensor in vitro (42) (Fig. 1 B). The maximal (Rmax) and minimal (Rmin) anisotropy values were 0.259 and 0.184 in 0 and 1 mM Ca^2+^, respectively. These results indicated that the Venus FLARE-Cameleon reporter was functionally expressed and responsive to changes in clamped Ca^2+^ concentration.

The in situ calibration values were next used to calculate Ca^2+^ concentrations from images obtained during peak and trough of the circadian cycle. Anisotropy values were calculated from all pixels, in all cells within the imaging region. Each image typically contained between two and five neurons expressing Venus FLARE-Cameleon after image thresholding. Average anisotropies obtained during the peak (0.223 ± 0.002) were significantly lower compared to anisotropies obtained during the trough (0.228 ± 0.002), indicating that more Ca^2+^ was bound to the sensor during the peak of the circadian cycle (Fig. 1 C). This corresponded to an estimated Ca^2+^ concentration that was 1.3-fold higher at the peak (218 ± 16 nM;range 53–408 nM) compared to the trough (172 ± 13 nM; range 21–365 nM) (Fig. 1 D). These data show that a circadian rhythm in intracellular Ca^2+^ can be detected from SCN neurons with the Venus FLARE-Cameleon sensor, and to our knowledge, provides a new method to track circadian changes in cytosolic Ca^2+^.

To define the response of the biosensor to acute changes in Ca^2+^ signaling under these in situ conditions, 50 mM KCl was applied to a subset of SCN slices at the peak and trough. Volumetric images were acquired in 10-s intervals for a 2-min baseline period before KCl was applied to the bath chamber and up to 10 min after KCl application. Consistent with prior studies (67), SCN slices responded to KCl treatment with a transient decrease in anisotropy values (peak: −0.024 ± 0.005; trough: −0.018 ± 0.003), corresponding to a transient increase in neuronal Ca^2+^ (peak: +271 ± 77 nM; trough: +524 ± 212 nM). The maximal KCl-evoked responses were compared with the average baseline anisotropies and Ca^2+^ concentrations at the circadian peak and trough. KCl produced a transient increase in Ca^2+^ levels of 3.66-fold during the peak (baseline: 102 ± 25 nM, KCl: 373 ± 78 nM; n = 5) and 3.52-fold during the trough (baseline: 208 ± 20 nM, KCl: 732 ± 228 nM; n = 8). Thus, Venus FLARE-Cameleon detects changes in Ca^2+^ evoked during maximal Ca^2+^ signaling at both the peak and trough.

**FIGURE 2:**
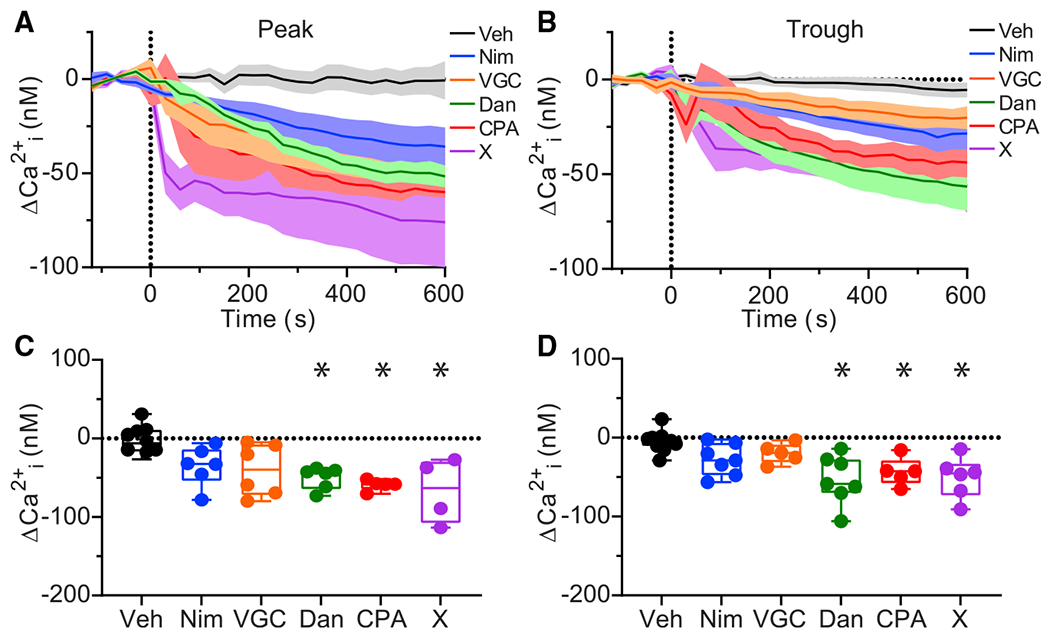
Effects of Ca^2+^ channel inhibitors on peak and trough Ca^2+^ concentration. (*A* and *B*) Time course of the change in Ca^2+^ concentration (ΔCa^2+^ = Ca^2+^ – average Ca^2+^ from 2-min baseline) before and after the application of vehicle control (Veh) or Ca^2+^ channel inhibitors at the peak (*A*) and trough (*B*). Data are mean ± SEM. (*C* and *D*) Plots of median, 25th and 75th percentile (*boxes*), and minimal and maximal (*whiskers*) changes in Ca^2+^ concentration for individual slices quantified from 9 to 10 min after drugs were applied at the peak (*C*) and trough (*D*). Inhibition of L-type Ca^2+^ channels with nimodipine (Nim, 10 *μ*M) or inhibition of N/P/Q/R/T-type Ca^2+^ channels with VGC (a mixture of 3 *μ*M ConoGVIA, 200 nM AgaIVA, 30 *μ*M nickel, and 1 *μ*M TTA-P2) did not significantly affect peak or trough Ca^2+^ levels compared to Veh. Inhibition of ryanodine receptors with dantrolene (Dan, 10 *μ*M), inhibition of SERCA-ATPase with cyclopiazonic acid (CPA, 10 *μ*M) and combined inhibition of voltage-gated Ca^2+^ channels and ryanodine receptors with a cocktail containing Dan, Nim, and VGC (cocktail X) significantly decreased Ca^2+^ at peak and trough. *p < 0.05, one-way ANOVA and Bonferroni post hoc test between drug and vehicle control conditions at peak (Nim, *p* = 0.3; VGC, *p* = 0.1; Dan, *p* = 0.02; CPA, *p* = 0.005; X, *p* = 0.003) and trough (Nim, *p* = 0.4; VGC, *p* = 1; Dan, *p* = 0.0004; CPA, *p* = 0.02; X, *p* = 0.002). Post hoc values were *p* > 0.05 for all other comparisons. Data points represent average measurements from individual SCN slices (one imaging region per slice). N = 4–11 slices per condition.

Next, to test the contributions of the different voltage-gated and intracellular Ca^2+^ channel subtypes to neuronal Ca^2+^ in the SCN, we measured the effects of Ca^2+^ channel inhibitors on anisotropy (Fig. S3, A–D) and estimated Ca^2+^ concentration (Fig. 2, A–D) during the peak and trough of the circadian cycle. For Ca^2+^ channel pharmacology experiments, volumetric images were captured in 30-s intervals for 2 min to obtain baseline anisotropy values before drugs or vehicle controls were applied. The effects of each drug were analyzed in slices imaged in 30-s intervals for 2 min of baseline and for 10 min after the application of a drug or vehicle control (Veh, <0.1% DMSO), which were added to the bath solution just before time 0. The average change in anisotropy values (Fig. S3, A and B) and corresponding change in Ca^2+^ concentrations (Fig. 2, A and B) relative to the baseline average of each slice were plotted as a function of time. Anisotropy values were stable, with no significant change for the duration of the 2-min baseline recordings in each condition (Fig. S3, A and B) (*p* = 0.3, two-way repeated-measures ANOVA). As a control for neuronal health after drug treatments, 50 mM KCl was applied to some slices after drug effects were obtained. KCl produced decreases in anisotropy values corresponding to increases in Ca^2+^ concentration that were 2–20 times higher than baseline Ca^2+^ levels (data not shown). The duration and magnitudes of these transient KCl-evoked responses were similar to those observed for slices in control conditions. These KCl responses obtained at the end of the experimental protocol verify that SCN slices are able respond to stimuli after Ca^2+^ channel inhibitors were applied, demonstrating that drug exposure did not affect slice viability.

First, to probe the contributions of voltage-gated channels, we used 10 *μ*M Nim to target L-type Ca^2+^ channels and a cocktail containing voltage-gated channel inhibitors (VGCs) targeting N-type (3 *μ*M ConoGVIA), P/Q-type (200 nM AgaIVA), R-type (30 *μ*M nickel), and T-type (1 *μ*M TTA-P2) Ca^2+^ channels. For each slice, paired comparisons were made between baseline, and the Ca^2+^ concentration averaged from 9 to 10 min after drugs were applied (Fig. S3, C–F). Application of vehicle control (Veh, <0.1% DMSO) during the peak or the trough did not significantly affect anisotropy values (Fig. S3, C and D) or Ca^2+^ concentrations (Fig. S3, E and F) compared to baseline. In contrast, voltage-gated Ca^2+^ channel inhibitors produced an increase in anisotropy values (Fig. S3, C and D), which corresponded to a reduction in the Ca^2+^ levels in paired comparison to baseline values (Fig. S3, E and F). These data implicate voltage-gated Ca^2+^ channels as contributors to the cytosolic Ca^2+^ levels during the peak and the trough.

We then tested the contributions of intracellular Ca^2+^ channels. Previous studies have shown that inhibiting RyR-mediated Ca^2+^ release from the ER produced a decrease in cytosolic Ca^2+^ levels (24,41), but the effect of inhibiting intracellular Ca^2+^ channels at both the peak and trough has not been systematically tested. 10 *μ*M Dan was used to inhibit RyRs, and 10 *μ*M CPA was used to target the SERCA-ATPase, which inhibits refilling of ER Ca^2+^ stores. CPA produces ER store depletion and subsequent inhibition of both RyR-and IP_3_R-mediated Ca^2+^ release (68). In a third condition, a cocktail (X) containing a combination of the VGCs, plus nimodipine and dantrolene to collectively inhibit RyRs along with the voltage-gated channels, was applied. Application of cocktail X thus inhibits other channels without blocking IP_3_Rs. We found that each inhibitor of intracellular Ca^2+^ channels produced a significant decrease in Ca^2+^ levels at the peak and the trough in a paired comparison to the baseline values for each slice (Fig. S3, E and F). These results suggest that intracellular channels also contribute to cytosolic Ca^2+^ levels at both the peak and the trough.

Determination of the relative contributions for VGCCs and intracellular Ca^2+^ channels across experiments requires accounting for the variation in baseline Ca^2+^ levels in each SCN slice. To make this comparison, the average Ca^2+^ concentration from 2 min of baseline was subtracted from the average Ca^2+^ levels 9–10 min after drug application to obtain the change in Ca^2+^ (ΔCa^2+^) within each slice. First, we focused on the ΔCa^2+^ produced by each drug during the peak (Fig. 2, A and C). Starting with the vehicle controls, the ΔCa^2+^ was negligible at −2 ± 7 nM (−2 ± 5% change) (Fig. 2 C). The voltage-gated channel inhibitors produced a ΔCa^2+^ of −35 ± 10 nM (−20 ± 4% change) (Nim) and −40 ± 13 nM (−19 ± 4%) (VGC);however, these decreases were not statistically different than the vehicle control. This may be partly explained by the variability in the ΔCa^2+^ responses of individual slices, which ranged from −6 to −78 nM (Nim) and −5 to −79 nM (VGC) (Fig. 2 C). In contrast, intracellular Ca^2+^ channel inhibitors produced decreases in peak Ca^2+^ that were significantly larger than the vehicle controls. Dantrolene produced the largest decrease, with a ΔCa^2+^ of −50 ± 6 nM (−36 ± 6% change). Similarly, the SERCA inhibitor CPA produced a ΔCa^2+^ of −59 ± 3 nM (−24 ± 5%), and the mixture of VGC inhibitors along with nimodipine and dantrolene added together (X cocktail) produced a ΔCa^2+^ of −67 ± 21 nM (−22 ± 6%) (Fig. 2 C). The responses of individual slices to intracellular Ca^2+^ channel inhibitors were less variable, with the range of −38 to −73 nM for dantrolene, −52 to −71 nM for CPA, and −27 to −114 nM for cocktail X. Taken together, these results suggest that peak intracellular Ca^2+^ is predominantly set by RyR channel contribution.

These inhibitors had similar effects on Ca^2+^ during the trough of the circadian cycle (Fig. 2 D). Vehicle control had little effect, −5 ± 4 nM (−7 ± 5% change). Nimodipine decreased Ca^2+^ by −28 ± 8 nM (−17 ± 5%) overall with a range of responses between −2 and −56 nM. Similarly, VGC decreased Ca^2+^ by −20 ± 6 nM (−16 ± 3%), with changes in Ca^2+^ ranging from −3.5 to −37 nM. The decreases in trough Ca^2+^ were significantly larger for dantrolene, −53 ± 12 nM (−28 ± 6%); CPA, −43 ± 8 nM (−32 ± 7%); and cocktail X, −51 ± 11 nM (−23 ± 5%). These overall decreases were accompanied by a larger range in ΔCa^2+^ responses, which were −14 to −106 nM for dantrolene, −16 to −65 nM for CPA, and −15 to −91 nM for cocktail X (Fig. 2 D). Thus, at both peak and trough time points, the largest decrease in Ca^2+^ was produced by dantrolene. In contrast, the contribution of voltage-gated channels appears less significant and in some cases has more variability.

## DISCUSSION

A primary advance of this study, to our knowledge, is the novel application of Venus-cp172Venus FLARE-Cameleon sensor (42) with piSPIM (59) to measure biologically controlled changes in Ca^2+^ in live organotypic brain tissue. The Venus FLARE-Cameleon Ca^2+^ sensor captured the collective signal in basal cytosolic Ca^2+^ averaged from multiple neurons within a region of the SCN without integrating shorter-timescale Ca^2+^ signals, such as action-potential-evoked Ca^2+^ transients. Using these techniques, Ca^2+^ concentrations (218 ± 16 and 172 ± 13 nM, respectively) were refined over prior absolute and relativistic estimates (27). These SCN concentrations are consistent with basal Ca^2+^ levels typically measured in other neuronal cell types, ~40–190 nM (69), and the values reported using other ratiometric Ca^2+^ sensors in the SCN. In prior studies, peak values ranged between 50 and 440 nM, and trough values ranged between 50 and 150 nM (27). However, prior measurements using Fura-2 (13,29,41,70) or genetically encoded sensors such as Yellow Cameleons (24,25,33,71) did not utilize in situ calibration of the Ca^2+^ sensor. These measurements relied on cell-free in vitro calibrations, which does not account for factors in the intracellular environment that could affect the Ca^2+^ estimates (65). In this study, Ca^2+^ measurements were obtained using a Ca^2+^ sensor that was calibrated in SCN slices under the same experimental conditions at baseline and across different Ca^2+^ inhibitor experiments. Venus FLARE-Cameleon also has the added advantage of only occupying a single-color channel, which will allow imaging of multiple biosensors expressed in the same neuron. Thus, this study provides a foundation for future experiments to investigate the cross talk between Ca^2+^ and other cellular signaling components toward piecing together how the ensemble circadian clock mechanism functions at a cellular level.

A central feature of SCN neurons is that they express different properties depending on the time of the circadian cycle. SCN neurons exhibit a state of increased excitability and increased activation of voltage-gated Ca^2+^ channels during the day (peak of the cycle) and a state of decreased excitability at night (trough of the cycle), during which voltage-gated Ca^2+^ channel activity is reduced (3,4,38,72). Yet, it has remained unclear whether this daily increase in voltage-gated Ca^2+^ channel activity is involved in maintaining the circadian pattern in cytosolic Ca^2+^, which is also highest during the circadian peak. Furthermore, no single study has directly compared the contributions for these different Ca^2+^ channel types to cytosolic Ca^2+^ levels at both peak and trough of the circadian cycle in intact SCN slices. Prior studies measured the effects of inhibitors on Ca^2+^ levels only at a single time point or employed only a single Ca^2+^ channel inhibitor (24,28,30,41,67). As a result of these methodological discrepancies, the relative contributions of the Ca^2+^ channel subtypes at both times of the circadian cycle have been less than fully conclusive. For example, in studies measuring the Ca^2+^ rhythms from the whole SCN, inhibition of L-type voltage-gated Ca^2+^ channels with nimodipine reduced the magnitude of the day-night difference in Ca^2+^ levels (28,30). However, other studies found no effect of nimodipine on Ca^2+^ levels (24,73).

With direct comparison of the relative contributions for each Ca^2+^ source under equivalent experimental conditions, the results in this study support the current view that intracellular RyR Ca^2+^ channels are major contributors to the Ca^2+^ levels during both the peak and trough of the circadian cycle. Because the combined inhibition of voltage-gated channels and RyRs did not significantly decrease Ca^2+^ levels further compared to inhibiting RyRs alone, it suggests that RyR inhibition produced the majority of the decrease in Ca^2+^. Inhibiting the SERCA-ATPase, which prevents Ca^2+^ reuptake into the ER and leads to a depletion of ER stores (68), also did not produce a decrease in Ca^2+^ that was larger in magnitude than the decrease observed when inhibiting RyRs alone. This further suggests that IP_3_Rs, which also can mediate ER Ca^2+^ release, may have a lesser contribution to cytosolic Ca^2+^ compared to RyRs, although this was not tested directly because of a lack of selective IP_3_R inhibitors (74). This study corroborates prior reports of decreased peak Ca^2+^ with RyR inhibition (6,24,41). RyR2 messenger RNA (mRNA) and protein also exhibit a daytime peak in expression (38,75). However, it is unlikely that an expression-based mechanism would fully account for the circadian oscillation in Ca^2+^ levels, as RyR activity is regulated by increases in intracellular Ca^2+^ (27). Although it remains to be determined whether other VGCCs contribute to Ca^2+^-induced Ca^2+^ release in SCN neurons, the lesser effect of inhibiting these channels suggests they do not serve as the primary sensors for RyR-mediated Ca^2+^ release. Other sources of calcium in the SCN not tested here include ionotropic glutamate receptors, including N-methyl-D-aspartate receptors and *α*-amino-3-hydroxy-5-methyl-4-isoxazolepropionic acid receptors (20). Ca^2+^ homeostasis is also maintained by the activity of Na^+^-Ca^2+^ exchanger types 1 and 2 (NCX1 and NCX2) (38,75) and endoplasmic reticulum Ca^2+^-ATPases (SERCA) (70), which mediate Ca^2+^ efflux or uptake into ER stores, whereas uptake of Ca^2+^ via mitochondrial NCX (76) and Ca^2+^-binding proteins buffer cytosolic Ca^2+^ (77–79). There is also potential involvement of store-operated Ca^2+^ entry channels (80). Another mechanism that could be involved in regulating basal Ca^2+^ over the circadian cycle, in conjunction with the activity of ion channels, is Ca^2+^ buffering by Ca^2+^-binding proteins (69), which can alter basal Ca^2+^ as well as influence the amplitude and decay of stimulus-evoked Ca^2+^ transients (81,82), leading to changes in firing properties of neurons (69). SCN neurons express calbindin D_28K_ and calretinin (77,83). In SCN, levels of cytosolic calbindin protein have been observed to change over the course of the circadian cycle (77). This evidence suggests that these Ca^2+^ binding proteins can be regulated based on the daily requirements of SCN neurons and could play an important role in circadian Ca^2+^ rhythms. Further studies will be required to investigate their contributions.

This study did not focus on defining the Ca^2+^ regulatory mechanisms by subregion, but the phase of the Ca^2+^ rhythm exhibits regional differences. It has not yet been addressed whether the Ca^2+^ channels themselves differ by subregion as the basis. The shell region of the SCN, defined by expression of the neuropeptide arginine vasopressin, exhibits a rhythmic Ca^2+^ peak 3–5 h before the core, defined by vasoactive intestinal polypeptide (VIP) expression (28,30,33). Shell Ca^2+^ rhythms also had higher amplitudes. These regional phase and amplitude differences could contribute to the wide variation in Ca^2+^ values observed in the baseline measurements of this study, as the ROIs were located in the center of the SCN and undefined with respect to the core and shell boundaries. In addition, sequential application of inhibitors could reveal whether the relative contribution of Ca^2+^ channel types differs between subregions. However, in previous studies, at least one inhibitor (nimodipine) failed to show a regional difference in its effects on Ca^2+^ levels (30), leaving open the question of which Ca^2+^ channels produce these phase differences. Regional differences are also present in *Drosophila* clock neurons (84,85), in which basal Ca^2+^ levels were sensitive to RNA interference (RNAi) knockdown of intracellular Ca^2+^ release via IP_3_Rs or SERCA, but only one set of neurons was sensitive to VGCC knockdown (86).

## Supplementary Material

1
