# Supplementary material for: Comparative Ca2+ channel contributions to intracellular Ca2+ levels in the circadian clock
Source: Biophys Rep (N Y). 2021 Jul 21;1(1):100005. doi: 10.1016/j.bpr.2021.100005 (PMC8942421; doi:10.1016/j.bpr.2021.100005)
Supplement: Document S1. Supporting materials and methods and Figs. S1–S3 [file mmc1.pdf]

Biophysical Reports, Volume *1*

Supplemental information

Comparative Ca^{2+} channel contributions to intracellular Ca^{2+} levels in the circadian clock

Amber E. Plante, Vishnu P. Rao, Megan A. Rizzo, and Andrea L. Meredith

Supporting Material

Comparative Ca^{2+} channel contributions to intracellular Ca^{2+} levels in the circadian clock

Amber E. Plante, Vishnu P. Rao, Megan A. Rizzo and Andrea L. Meredith
Dept. of Physiology, University of Maryland School of Medicine, Baltimore, MD, 21201

SUPPLEMENTAL MATERIALS AND METHODS

Organotypic slices cultured on MEAs

For confocal imaging experiments, freshly dissected SCN slices were plated onto MEA probes (Alpha MED Scientific Inc., Osaka, Japan; MED-P210A) that were pre-treated overnight with polyethyleneimine (0.1% w/v, Millipore Sigma; P3143) in borate buffer solution (25 mM, pH 8.0, Millipore Sigma; S9640) and rinsed with sterile water as per the manufacturer's instructions (Alpha MED Scientific Inc.; available at https://www.med64.com/documentation). MEA probe dishes were placed in 100 mm petri dishes, surrounded by ~7 mL sterile water in a humidified incubator (37°C, 5% CO_2). Slices were maintained in 200-300 µL culture medium. Medium (50%) was exchanged every ~48 hours for the first 8 days in culture. On culture day 3, SCN slices cultured on MEAs were transduced with 0.3 µL of AAV1 containing GCaMP6f cDNA expressed under the hSyn1 promoter (AAV1.Syn1.GCaMP6f.SV40.WPRE; stock $1x10^{13}$ vg/mL, Addgene, #100837-AAV1) (Figure S1A) and cultured for an additional 14-21 days. On culture day 10 and thereafter, MEA dishes were sealed with vacuum grease and glass coverslips and 100% of the medium was exchanged every 72 hours.

MEA activity recording and analysis

Circadian rhythms in action potential frequency were recorded from SCN slices on MEAs between culture days 14–21 using a MED64-Plex8 system (Alpha MED Scientific Inc.) as described previously (1). Spontaneous action potential activity was acquired for 3 full circadian cycles (72 hours) in 5-second sweeps every 5 min, with setting low cut frequency 100 Hz and high cut frequency 10,000 Hz (Mobius vWin7, Alpha MED Scientific Inc.). Firing frequency was determined from each 5-second sweep using threshold-based counting of downward-spiking action potentials at ~1.5× the level of baseline noise (typically between −10 and −25 µV). Raw action potential frequencies from 3 circadian cycles from all electrodes visually identified to be within the boundaries of the SCN were plotted in Microsoft Excel. Activity recordings were considered rhythmic if the raw action potential frequency peaked once every ~24 hours with a maximum frequency ≥ 3 Hz compared to the minimum (trough) frequency. Rhythmic recordings from 3-5 electrodes were smoothed with a 6-hour moving-window average and used to calculate an average peak and trough time for each SCN slice (Figure S1).

Confocal imaging of GCaMP fluorescence

On culture day 10, 100% of the culture medium was exchanged and MEA recordings were performed for 3 days (culture days 10-13) to obtain average peak and trough times in action potential activity for each slice. Between culture days 14-21, MEA recordings were repeated for 3 days. During this recording period, MEA probes were temporarily removed for confocal imaging in 30-minute time windows at each peak and trough timepoint to obtain circadian action potential and GCaMP6f Ca^{2+} measurements from the same SCN slice (Figure S1B). Culture

medium exchanges were performed 12 hours prior to conducting simultaneous MEA recording and Ca^{2+} imaging experiments. Fluorescence imaging was performed using a W1 spinning disk confocal on a Ti2 inverted microscope (Nikon) housed at the University of Maryland School of Medicine confocal facility. Probes were temporarily removed from recording headstages during the peak and trough of action potential firing and placed in an environmentally controlled microscope chamber at 37°C with 5% CO_2 for confocal imaging to obtain circadian signals in GCaMP6f fluorescence from two full circadian cycles (three troughs and two peaks). GCaMP6f fluorescence was excited for 100 ms with a 488 nm laser and collected with a 515-149 nm emission filter cube. Images (12-bit, 324 nm pixel width and height, no binning) were acquired with a 20× air objective (Nikon) at 10 fps for a duration of 10 seconds and saved as Tiff stack files. LED light intensity was set at 20 to 40% of maximal level, depending on GCaMP6f expression levels.

Confocal image processing and data analysis

GCaMP6f images were converted to 8-bit greyscale Tiff files, stacked and aligned in ImageJ (FIJI) (2) using the StackReg plugin (FIJI) (3). Fluorescence intensities from individual neurons within the boundaries of the SCN were measured using oval regions of interest (ROIs) that were manually drawn in ImageJ (FIJI) within the somatic region of individual cells excluding the nucleus, since GCaMP6f expression is cytosolic (4) (Figure S1A). Neurons with apparent baseline fluorescence at least 100× greater than the background fluorescence were used for analysis. For each SCN slice, a minimum of 6 ROIs were drawn in regions devoid of cells and used for background fluorescence measurements. A minimum of 8 cells (one ROI per cell) were analyzed per SCN slice. The average of the mean background fluorescence from 6 ROIs (BGD) was subtracted from the integrated density of the cell ROI fluorescence (Fcell) using the equation: Fcell – (area of ROI × BGD) in Microsoft Excel. Background-subtracted fluorescence intensities for each cell were plotted over time and normalized by dividing the average corrected fluorescence at each timepoint by the maximum value (F/Fmax) of that cell. To calculate the overall F/Fmax for each slice, F/Fmax values from individual cells were averaged within each timepoint, resulting in one F/Fmax value per slice at each timepoint for a total of five values per slice.

SUPPLEMENTAL FIGURES

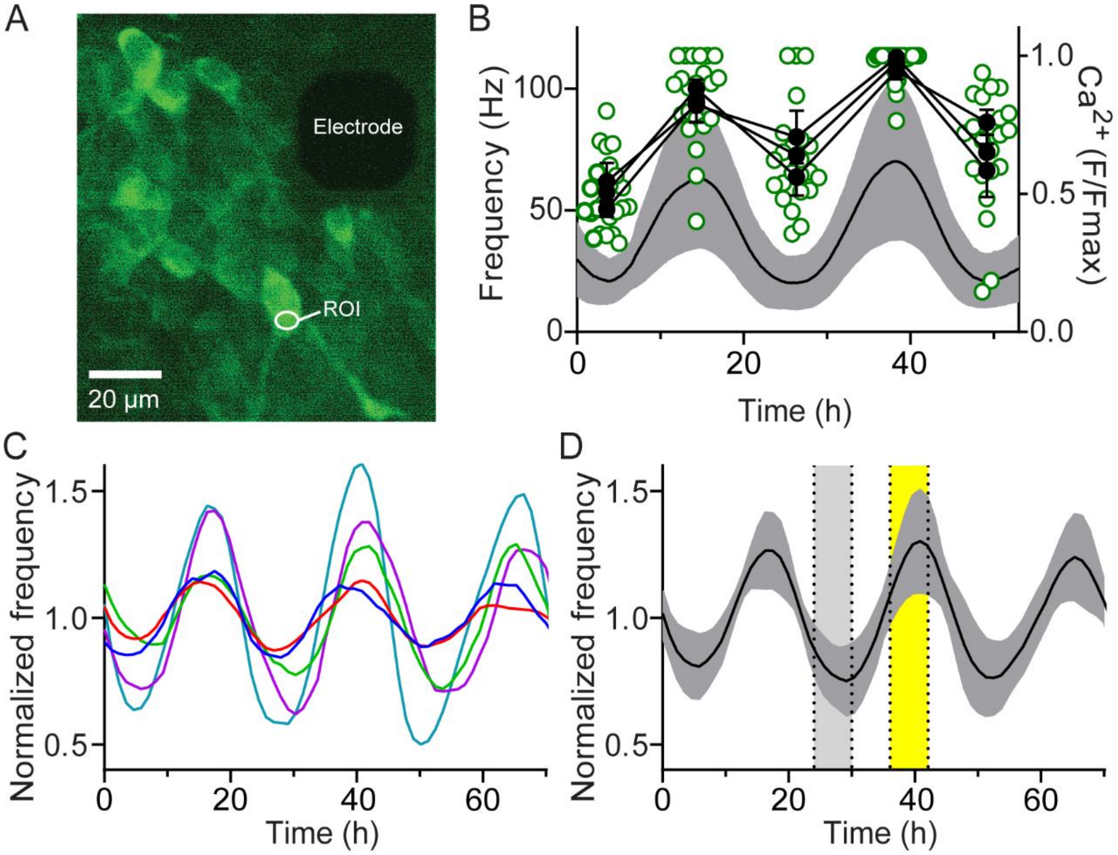

Figure S1. Establishment of the phase for circadian rhythms in Ca^{2+} and action potential firing. SCN organotypic slices were transfected with the fluorescent GCaMP6f Ca^{2+} sensor, targeted to neurons using the human synapsin1 (hSyn1) promoter (5). For each slice, action potential firing was recorded for 3 days by multi-electrode array to determine whether slices were rhythmic. The circadian peak and trough were determined from the firing activity rhythm for each slice. Using these as phase reference points, confocal imaging was performed in 30-minute time windows to assess the circadian pattern in intracellular Ca^{2+}.

A) Confocal image of GCaMP6f Ca^{2+} sensor fluorescence in neurons from an SCN slice cultured on a multielectrode array at 20× magnification with example of a somatic region of interest (ROI) used for analysis in a single neuron (white circle).

B) Peak and trough Ca^{2+} measurements superimposed on action potential rhythms, showing Ca^{2+} signals are higher at the peak of firing compared to the trough of firing. This control demonstrates that under these culturing and imaging conditions, the underlying Ca^{2+} signals were rhythmic and diurnal, as prior studies have shown (6-8). Data plots are mean action potential frequency ± SD (black line with grey shading, left axis) recorded from slices on MEAs overlayed with GCaMP6f fluorescence normalized to the maximum (F/Fmax, right axis) measured from the same SCN slices over 3 days. GCaMP6f Ca^{2+} signals from individual neurons (open green circles) and the mean Ca^{2+} signal ± SEM of individual SCN slices (closed black circles) at each timepoint are shown. Ca^{2+} was significantly higher during the peak in action potential firing compared to the trough ($P < 0.0001$, two-way repeated measures ANOVA with Bonferroni *post hoc* test). (N = 3 slices, 8 neurons per slice)

C) Circadian rhythms in action potential firing exhibited synchronized phases across the slices in culture, with average peaks and troughs occurring at approximately the same time of the circadian cycle. The firing rate rhythm phases had a standard deviation of 0.7 hours (N = 5 slices). Data plots show action potential firing recorded from SCN slices expressing Venus FLARE-Cameleon on MEAs plotted as frequency smoothed with a 6-h moving window average (normalized to a 24-hr moving window average of raw frequency values) from individual SCN slices (colored lines).

D) Since Ca^{2+} rhythms have been reported to peak ~0-5 hours before the peak in action potential firing (27), the indicated 6-hour time window surrounding the average peak and trough times in action potential firing were identified to perform the 30-minute piSPIM imaging experiments detailed in the main text. Data plots are mean action potential frequency ± SD (black line and grey shading) from panel C overlayed with the 6-h time windows at the peak (yellow bar) and trough (grey bar).

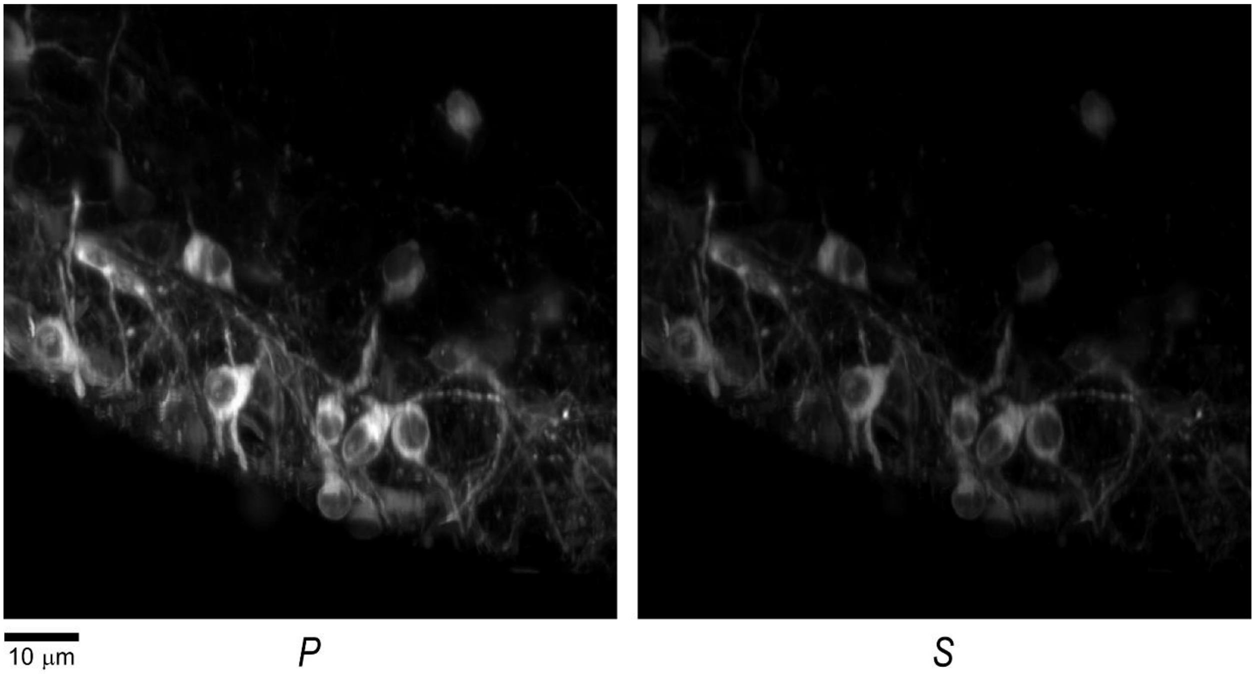

Figure S2. Three-dimensional projections of piSPIM data. SCN neurons expressing the Venus-cp172Venus FLARE-Cameleon biosensor. Images represent parallel (*P*) and perpendicular (*S*) z-projections from a representative 20-µm volumetric image stack used for calcium quantification. 3D projections were made using FIJI.

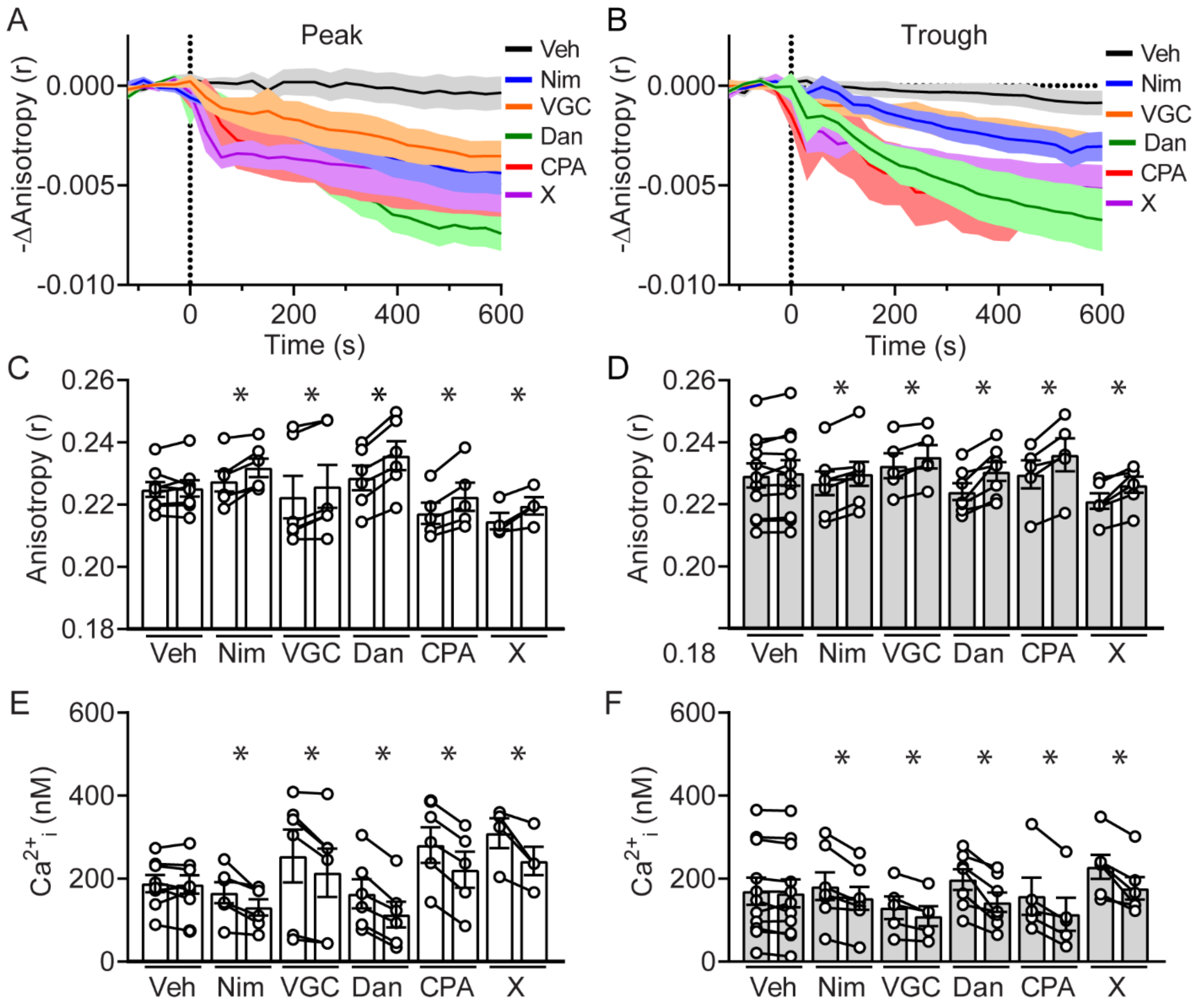

Figure S3. Effects of Ca^{2+} channel inhibitors on peak and trough anisotropy and Ca^{2+} concentration. Anisotropy and Ca^{2+} concentrations for data in main Figure 2.

A, B) Time course of the negative change in anisotropy (-Δanisotropy = -1 × (anisotropy – average anisotropy from 2 min baseline)) before and after application of Ca^{2+} channel inhibitors at peak (A) and trough (B).

C, D) Anisotropy values averaged from baseline recordings and from 9-10 min after application of Ca^{2+} channel inhibitors for each slice at peak (C) or trough (D).

E, F) Average Ca^{2+} concentrations at baseline and after application of Ca^{2+} channel inhibitors at peak (D) and trough (F) calculated from anisotropies in C and D. Compared to baseline, anisotropy was increased and Ca^{2+} was decreased by each Ca^{2+} channel inhibitor. *, Significance ($P < 0.05$) tested with paired Student's *t* tests for anisotropy values at peak (Veh, P =0.7; Nim, P = 0.01; VGC, P = 0.007; Dan, P = 0.0005; CPA, P = 0.001; X, P = 0.04) and trough (Veh, P = 0.2; Nim, P = 0.004; VGC, P = 0.01; Dan, P =0.005; CPA, P = 0.008; X, P = 0.009) and for Ca^{2+} concentrations at peak (Veh, P = 0.7; Nim, P = 0.3; VGC, P = 0.1; Dan, P = 0.02; CPA, P = 0.005; X, P = 0.003) and trough (Veh, P = 0.2; Nim, P = 0.01; VGC, P = 0.02; Dan, P =0.004; CPA, P = 0.006; X, P = 0.007). Data are plotted as mean ± SEM. Data points represent paired recordings from individual SCN slices (one imaging region per slice). N= 4-11 slices per drug condition.

SUPPLEMENTAL REFERENCES

1. McNally, B. A., A. E. Plante, and A. L. Meredith. 2019. Diurnal properties of voltage-gated Ca^{2+} currents in suprachiasmatic nucleus and roles in action potential firing. *J Physiol* 598(9):1775–1790.

2. Schindelin, J., I. Arganda-Carreras, E. Frise, V. Kaynig, M. Longair, T. Pietzsch, S. Preibisch, C. Rueden, S. Saalfeld, B. Schmid, J. Y. Tinevez, D. J. White, V. Hartenstein, K. Eliceiri, P. Tomancak, and A. Cardona. 2012. Fiji: an open-source platform for biological-image analysis. *Nat Methods* 9(7):676-682.

3. Thevenaz, P., U. E. Ruttimann, and M. Unser. 1998. A pyramid approach to subpixel registration based on intensity. *IEEE Trans Image Process* 7(1):27-41.

4. Chen, T. W., T. J. Wardill, Y. Sun, S. R. Pulver, S. L. Renninger, A. Baohan, E. R. Schreiter, R. A. Kerr, M. B. Orger, V. Jayaraman, L. L. Looger, K. Svoboda, and D. S. Kim. 2013. Ultrasensitive fluorescent proteins for imaging neuronal activity. *Nature* 499(7458):295-300.

5. Kugler, S., E. Kilic, and M. Bahr. 2003. Human synapsin 1 gene promoter confers highly neuron-specific long-term transgene expression from an adenoviral vector in the adult rat brain depending on the transduced area. *Gene Ther* 10(4):337-347.

6. Ikeda, M., T. Sugiyama, C. S. Wallace, H. S. Gompf, T. Yoshioka, A. Miyawaki, and C. N. Allen. 2003. Circadian dynamics of cytosolic and nuclear Ca^{2+} in single suprachiasmatic nucleus neurons. *Neuron* 38(2):253-263.

7. Jones, J. R., T. Simon, L. Lones, and E. D. Herzog. 2018. SCN VIP neurons are essential for normal light-mediated resetting of the circadian system. *J Neurosci* 38(37):7986-7995.

8. Enoki, R., D. Ono, S. Kuroda, S. Honma, and K. I. Honma. 2017. Dual origins of the intracellular circadian calcium rhythm in the suprachiasmatic nucleus. *Sci Rep* 7:41733.
