# Supplementary material for: Comparative Ca2+ channel contributions to intracellular Ca2+ levels in the circadian clock
Source: Biophys Rep (N Y). 2021 Jul 21;1(1):100005. doi: 10.1016/j.bpr.2021.100005 (PMC8942421; doi:10.1016/j.bpr.2021.100005)
Supplement: Document S2. Article plus supporting material [file mmc2.pdf]

Biophysical Reports

ARTICLE

# Comparative $Ca^{2+}$ channel contributions to intracellular $Ca^{2+}$ levels in the circadian clock

Amber E. Plante,[1] Vishnu P. Rao,[1] Megan A. Rizzo,[1] and Andrea L. Meredith[1,*]
[1]Department of Physiology, University of Maryland School of Medicine, Baltimore, Maryland

ABSTRACT Circadian rhythms in mammals are coordinated by the central clock in the brain, located in the suprachiasmatic nucleus (SCN). Multiple molecular and cellular signals display a circadian variation within SCN neurons, including intracellular $Ca^{2+}$, but the mechanisms are not definitively established. SCN cytosolic $Ca^{2+}$ levels exhibit a peak during the day, when both action potential firing and $Ca^{2+}$ channel activity are increased, and are decreased at night, correlating with a reduction in firing rate. In this study, we employ a single-color fluorescence anisotropy reporter (FLARE), Venus FLARE-Cameleon, and polarization inverted selective-plane illumination microscopy to measure rhythmic changes in cytosolic $Ca^{2+}$ in SCN neurons. Using this technique, the $Ca^{2+}$ channel subtypes contributing to intracellular $Ca^{2+}$ at the peak and trough of the circadian cycle were assessed using a pharmacological approach with $Ca^{2+}$ channel inhibitors. Peak (218 $\pm$ 16 nM) and trough (172 $\pm$ 13 nM) $Ca^{2+}$ levels were quantified, indicating a 1.3-fold circadian variance in $Ca^{2+}$ concentration. Inhibition of ryanodine-receptor-mediated $Ca^{2+}$ release produced a larger relative decrease in cytosolic $Ca^{2+}$ at both time points compared to voltage-gated $Ca^{2+}$channels. These results support the hypothesis that circadian $Ca^{2+}$ rhythms in SCN neurons are predominantly driven by intracellular $Ca^{2+}$ channels, although not exclusively so. The study provides a foundation for future experiments to probe $Ca^{2+}$ signaling in a dynamic biological context using FLAREs.

## INTRODUCTION

$Ca^{2+}$ signaling is essential for the production of timekeeping signals in the mammalian circadian clock, which is located in the suprachiasmatic nucleus (SCN) of the hypothalamus. $Ca^{2+}$ is required for SCN neurons to maintain 24-h rhythms in clock gene expression (1,2) and action potential activity (3–6). These main SCN output signals ultimately control the timing of downstream physiological processes (7–9) and behaviors (10–12). $Ca^{2+}$ signaling is also required for SCN neurons to respond to external inputs (13–20) that lead to shifts in molecular (21), cellular (14,22), and behavioral rhythms (23).

A circadian pattern in intracellular free $Ca^{2+}$ has been identified in both SCN neurons and glia (24–29). SCN neurons exhibit a circadian rise in cytosolic $Ca^{2+}$ concentration that typically reaches a peak during the day, ~0–5 h before the peak in action potential firing, and is reduced at night (24,27,30–32). These rhythmic $Ca^{2+}$ signals can be observed in single SCN neurons as well as from whole SCN slices (19,24,28,30,32–35). However, the major $Ca^{2+}$ channels that drive these $Ca^{2+}$ rhythms are still under investigation.

Previous studies have implicated multiple $Ca^{2+}$ channel subtypes that contribute to $Ca^{2+}$ signaling in SCN neurons (27). Membrane depolarization stimulates $Ca^{2+}$ influx by activating voltage-gated $Ca^{2+}$ channels (VGCCs) including L-, N-, P/Q-, R-, and T-type channels (2–5). $Ca^{2+}$ release from intracellular stores in the endoplasmic reticulum (ER) is mediated by ryanodine receptors (RyR2 and RyR3) (24,36–38). $IP_3$ signaling stimulates $Ca^{2+}$ release from the ER by activating inositol 1,4,5-trisphosphate receptors ($IP_3Rs$) (2,39). Prior studies have shown that cytosolic $Ca^{2+}$ levels may be mediated in part by $Ca^{2+}$ release from intracellular ER stores, as pharmacological inhibition of RyRs produces a large decrease in cytoplasmic $Ca^{2+}$ (24,40,41). However, $Ca^{2+}$ influx through the plasma membrane from voltage-gated $Ca^{2+}$ channels may also contribute. Inhibition of L-type voltage-gated $Ca^{2+}$ channels with nimodipine or action potential firing with the $Na^{+}$ channel blocker tetrodotoxin partially reduce cytoplasmic $Ca^{2+}$ (40). To date, no single study has directly compared the contributions of the main

Submitted April 27, 2021, and accepted for publication July 8, 2021.
*Correspondence: ameredith@som.umaryland.edu
Editor: Shi-Wei Chu.
https://doi.org/10.1016/j.bpr.2021.100005
© 2021 The Author(s).
This is an open access article under the CC BY license (http://creativecommons.org/licenses/by/4.0/).

plasma membrane and intracellular $Ca^{2+}$ channels at both the peak and trough of the circadian cycle from intact SCN slices.

This study utilizes a newly developed fluorescent biosensor to provide a quantifiable and direct comparison for the contributions of voltage-gated and intracellular $Ca^{2+}$ channels to daytime (peak) and nighttime (trough) $Ca^{2+}$ levels in SCN neurons from intact brain slice cultures. Polarization inverted selective-plane illumination microscopy (piSPIM) was used to measure $Ca^{2+}$ concentration within SCN using a ratiometric, neuronally expressed $Ca^{2+}$ sensor, Venus FLARE-Cameleon (Venus-cp172Venus FLARE-Cameleon) (42). The Venus FLARE-Cameleon sensor is a fluorescence resonance energy transfer (FRET)-based fluorescence anisotropy reporter (FLARE) (42). $Ca^{2+}$ concentrations were estimated from in situ calibration of Venus FLARE-Cameleon fluorescence anisotropy signals in SCN slices. Pharmacological inhibitors targeting the major $Ca^{2+}$ channel subtypes were applied during the peak and trough phases of the diurnal cycle to evaluate the impact of different $Ca^{2+}$ sources on $Ca^{2+}$ levels. These data revealed a peak-to-trough difference in cytosolic $Ca^{2+}$ concentration that was higher during the day, with ryanodine receptors providing the largest contribution at both times of the diurnal cycle.

## MATERIALS AND METHODS

### Animals and ethical approval

Wild-type C57BL/6J mice were bred in a standard 12:12-h light-dark cycle. Male and female mice were killed for experiments via decapitation at postnatal day 4. All procedures involving mice were conducted in accordance with the University of Maryland School of Medicine Animal Care and Use Guidelines and approved by the Institutional Animal Care and Use Committee.

### Organotypic slice culture and viral transduction

Brains were dissected during the light cycle as described previously (3). Coronal sections of the hypothalamus (300 $\mu$m) were made on a manual tissue chopper (Stoelting, Wood Dale, IL) in ice-cold dissection medium containing bicarbonate-free Dulbecco's modified Eagle's medium (12100-046; Gibco, Gaithersburg, MD), 10 mM HEPES (pH 7.3), 100 U/mL penicillin/streptomycin (30-002-CI; MediaTech, Manassas, VA), and 2 mM L-glutamine (25-005-CI; MediaTech). Slices containing the SCN (one per animal) were cultured as organotypic interface explants (43).

For piSPIM experiments, SCN slices were plated onto filter membranes (PICM0RG50; Millipore Sigma, Burlington, MA) in 35-mm culture dishes (353001; Corning, Corning, NY) with 1.2 mL culture medium containing minimal essential medium (11095-080; Gibco), 25 mM HEPES (pH 7.3), 25% horse serum (16050-130; Gibco), 28 mM D-glucose (G8270; Millipore Sigma), 10 U/mL penicillin/streptomycin (30-002-CI; MediaTech), and 2 mM L-glutamine (25-005-CI; MediaTech). Cytosine $\beta$-D-arabinofuranoside (Ara-C, 20 $\mu$M; C6645; Millipore Sigma) was added to culture medium starting on culture day 2 to inhibit glial cell growth. Immediately after plating, slices were transduced with 1 $\mu$L of adeno-associated viral vector (AAV, serotype 1) containing Venus-cp172Venus FLARE-Cameleon biosensor DNA (42) (AAV1.hSyn1.Vencp172Ven Cameleon; stock $2.18 \times 10^{12}$ vg/mL; plasmid #pOTTC1612; Genetic Engineering and Viral Vector Core, National Institute on Drug Abuse, Baltimore, MD). Neuron-specific expression was driven by the human synapsin 1 (hSyn1) promoter (44). Slices were maintained for 14–21 days in a humidified incubator at 37°C (5% $CO_2$) with 100% of the culture medium exchanged every ~72 h.

A subset of slices transduced with Venus FLARE-Cameleon AAVs were plated onto multielectrode arrays on culture day 10 as described previously (43,45). SCN slices cultured on filters were excised from the surrounding filter and flipped (SCN side down) onto multielectrode arrays pretreated overnight with 500 $\mu$L of 0.1 mg/mL collagen (C8919; Sigma-Aldrich, St. Louis, MO) and maintained in culture medium as described in Supporting materials and methods.

### piSPIM imaging of fluorescence anisotropy

Imaging experiments were conducted in 6-h time windows using the peak and trough of firing rhythms as the reference point, in which images were obtained between 5 h before to 1 h after the time of the peak or the trough in action potential firing. Filter sections with SCN slices were excised, rinsed in phosphate-buffered saline, transferred to the microscope chamber, and equilibrated for 20–30 min in 6 mL of prewarmed imaging solution containing 125 mM NaCl, 8 mM NaOH, 5 mM KCl, 1 mM $MgCl_2$, 20 mM HEPES, 5 mM D-glucose, and 2.5 mM $CaCl_2$ (pH 7.20 $\pm$ 0.01 at 35°C). Fluorescence anisotropy imaging was performed on a polarization inverted selective-plane illumination microscopy (piSPIM) microscope with stage-scanning capability assembled and aligned as described previously (46–48). The collection arms of the microscope were fitted with filter wheels containing emission filters and an image splitting device, OptoSplit II (Cairn, Faversham, UK), to separate parallel (*P*) and perpendicular (*S*) polarizations. The microscope was housed in an environmentally controlled incubator (Okolab, Ambridge, PA) maintained at 37°C. Automated stage and piezo focus control hardware elements were purchased from Applied Scientific Instruments (Eugene, OR). Camera and piezo electronics were controlled using Micromanager software (available at https://micro-manager.org/) (49) on a Z840 workstation (Hewlett Packard, Palo Alta, CA). Volumetric images (16-bit grayscale) were collected on a Nikon Eclipse TE2000-U microscope with water-dipping objectives (MRD07420, 40×, 0.8 NA; Nikon, Tokyo, Japan) and a digital camera (Flashv4 Orca, C13440; Hamamatsu, Hamamatsu, Japan) as stack files with 20 image slices per volume (1-$\mu$m spacing, 512 $\times$ 1024 pixels per image slice, 332-nm pixel width and height, 2 $\times$ 2 binning). Samples were excited in 10-s (KCl experiments) or 30-s ($Ca^{2+}$ inhibitor experiments) intervals with a 488-nm laser. Images were collected from a ~170 $\times$ 340 $\times$ 20 $\mu$m area within the center of the SCN, which was visually identified under brightfield illumination at 4× magnification using the optic chiasm and third ventricle as reference landmarks. After baseline control images were acquired, imaging solution (100–200 $\mu$L) was removed from the bath, mixed with the appropriate amount of drug stocks or dimethyl sulfoxide (DMSO) (<0.01%, D2650; Sigma-Aldrich), and reapplied to the bath chamber. The temperature of the bath solution was 35 $\pm$ 0.1°C.

### piSPIM image processing and data analysis

Images were processed and analyzed according to Ross et al. (42,50) with some modifications using ImageJ (FIJI) macros and script executed in Python (v3.7). Volumetric image stacks were split to

separate $P$ and $S$ channels. Corresponding $P$ and $S$ image stacks ($512 \times 1024$ pixels) were aligned using a Python script and separated into individual images. The median grayscale value of the background intensities for each image was calculated and subtracted. An adaptive local thresholding method was used to obtain a binary clipping mask to separate cell signals from image background. The local threshold value for each pixel was calculated using the Gaussian-weighted sum of the neighborhood pixel intensities (51,52). Anisotropies ($r$) were calculated using pixel intensities above the threshold value from the corresponding background-subtracted $P$ and $S$ images using the equation (53)

$$r = \frac{P - gS}{P + 2gS}.$$

The g-factor constant ($g$) was measured using an isotropic fluorescein solution and calculated to account for the difference between $P$ and $S$ channel transmission efficiencies as previously described (50). The $r$-values for each image were summed across all images in each stack and plotted as a histogram distribution. A single mean $r$-value for each image stack was calculated with a Gaussian fit of the $r$ histogram distribution in Prism v8.4 (GraphPad Software, San Diego, CA). Scripts for automated image alignment, background subtraction, pixel thresholding, and $r$-value calculations were executed in Python.

## In situ calcium calibration

$Ca^{2+}$ buffering solutions were prepared using the method described in McGuigan et al. (54). To ensure EGTA concentrations in $Ca^{2+}$-EGTA and EGTA solutions were identical, a 2× EGTA stock solution containing all ingredients (except for NaOH and $CaCl_2$) was prepared and split into two volumes. $CaCl_2$ and NaOH were added to one volume and diluted to obtain a 1× $Ca^{2+}$-EGTA solution containing 125 mM NaCl, 44 mM NaOH, 5 mM KCl, 2 mM KOH, 1 mM $MgCl_2$, 20 mM HEPES, 1.8 mM 2-deoxy-D-glucose, 5 mM EGTA, 5 mM $CaCl_2$, 0.01 mM rotenone, 0.01 mM ionomycin, and 0.01 mM cyclopiazonic acid (CPA) (pH 7.20 $\pm$ 0.01 at 35°C). NaOH and HCl were added to the second volume to produce a final 1× EGTA (zero free $Ca^{2+}$) solution containing 125 mM NaCl, 44 mM NaOH, 5 mM KCl, 2 mM KOH, 1 mM $MgCl_2$, 20 mM HEPES, 1.8 mM 2-deoxy-D-glucose, 5 mM EGTA, 0.01 mM rotenone, 0.01 mM ionomycin, and 0.01 mM CPA (pH 7.20 $\pm$ 0.01 at 35°C). The appropriate quantities of $Ca^{2+}$-EGTA and EGTA solutions were mixed to obtain solutions with known free $Ca^{2+}$ concentrations calculated with WebMaxC standard (available online at https://somapp.ucdmc.ucdavis.edu/pharmacology/bers/maxchelator/webmaxc/webmaxcS.htm). SCN slices were equilibrated in $Ca^{2+}$ buffer solutions at least 20 min before imaging. All imaging solutions were prepared with $Ca^{2+}$-free liquid-chromatography mass-spectrometry (LC-MS)-grade water (WX0001-6; Sigma-Aldrich). The dissociation constant ($K_d$) and Hill coefficient ($n$) were determined by fitting a plot of the $r$ vs. $Ca^{2+}$ concentration data in Prism (GraphPad Software) with the equation

$$r = rmin + \frac{rmax - rmin}{1 + \left(\frac{K_d}{[Ca^{2+}]}\right)^n}.$$

## Statistics

Statistical tests were performed in Prism v8.4 (Graphpad Software). Changes in anisotropy values across baseline time points were tested with a two-way analysis of variance (ANOVA) with repeated measures. Student's $t$-tests (two tailed) were used to determine significant differences in anisotropy values and $Ca^{2+}$ concentrations between peak and trough time points. One-way ANOVA with Bonferroni's post hoc tests were used to determine significant differences in $\Delta Ca^{2+}$ between conditions at each time point. Paired $t$-tests (two tailed) were used to test for changes in anisotropy and $Ca^{2+}$ concentration between baseline and drug conditions for individual SCN slices at each condition. Significant differences in GCaMP6f fluorescence across multiple peak and trough time points were tested with a two-way, repeated-measures ANOVA and Bonferroni's post hoc test using the F/Fmax-values from individual cells for all slices across time points.

## Pharmacology

Pharmacological reagents were used at final concentrations of 10 $\mu$M nimodipine (Nim; N150; Alomone Labs, Jerusalem, Israel), 10 $\mu$M dantrolene (Dan; D9175; Sigma-Aldrich), 30 $\mu$M CPA (C-750; Alomone Labs), 3 $\mu$M $\omega$-conotoxin GVIA (ConoGVIA; C-300; Alomone Labs), 200 nM $\omega$-agatoxin IVA (AgaIVA; STA-500; Alomone Labs), 30 $\mu$M $NiCl_2$ ($Ni^{2+}$; 22387; Sigma-Aldrich), 10 $\mu$M ionomycin (407951; Sigma-Aldrich), 10 $\mu$M rotenone (R8875; Sigma-Aldrich), and 50 mM KCl (P9333; Sigma-Aldrich). Reagent stocks (1000×) were prepared in DMSO (Nim, Dan, CPA, ionomycin, rotenone) or water (ConoGVIA, AgaIVA, $Ni^{2+}$) and stored at $-20$°C. KCl was prepared as a 4 M HEPES-buffered stock solution.

# RESULTS

In the ex vivo organotypic slice preparation, the isolated SCN exhibits intrinsic circadian rhythmicity. First, rhythms in long-term spontaneous action potential activity were recorded by multielectrode array (Supporting materials and methods) to verify robust intrinsic circadian rhythms in cultured SCN slices before imaging. After establishing the diurnal phase using action potential firing, standard confocal imaging was used to verify intracellular $Ca^{2+}$ was also rhythmic under these experimental conditions using the $Ca^{2+}$ sensor GCaMP6f (Fig. S1). These data were then used to determine the time windows for quantitative $Ca^{2+}$ imaging using piSPIM.

To measure intracellular $Ca^{2+}$ using Venus FLARE-Cameleon (42), SCN slices were cultured on filter membranes (Fig. 1 *A i*) and transduced with AAVs containing Venus FLARE-Cameleon cDNAs (42) expressed under the neuron-specific hSyn1 promoter (44). $Ca^{2+}$ binding to the Venus FLARE-Cameleon protein induces FRET between the two Venus fluorophores (55–59), which is detected as a decrease in the polarization (anisotropy) of emitted light from the sensor (42,53,59–61). FRET-based measurements from Venus FLARE-Cameleon provide a ratiometric quantification of $Ca^{2+}$ concentration that is insensitive to variation in expression levels, cell morphology, illumination, or experimental preparations. Thus, this biosensor circumvents the variability in measurements that are based on fluorescence intensity (42,53,55,56,59,62,63,64), enabling quantitative

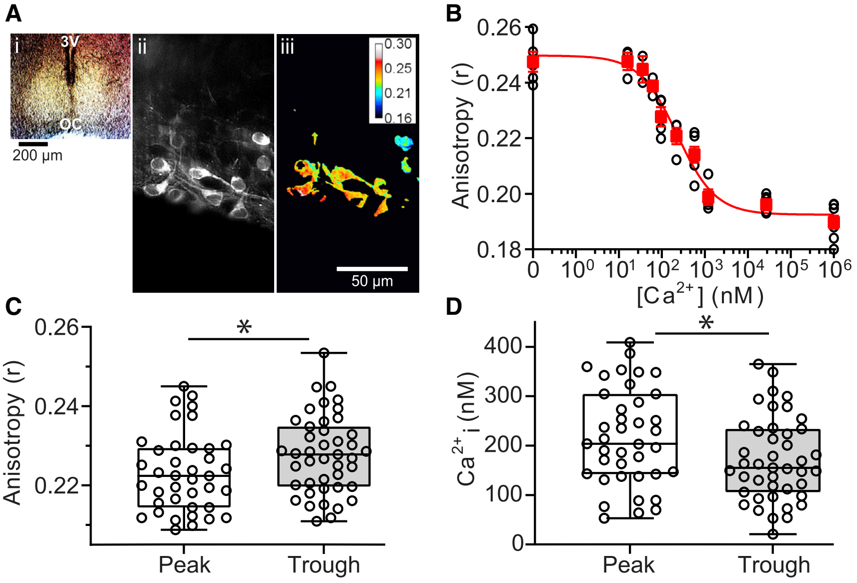


FIGURE 1 Neuronal $Ca^{2+}$ concentrations at peak and trough in SCN. (*A i*) Brightfield image of SCN at 4× magnification. (*A ii*) piSPIM image of polarized fluorescence signals from SCN neurons expressing Venus FLARE-Cameleon at 40× magnification. (*A iii*) Pseudocolor map of anisotropy values from the image in (*A ii*) calculated from parallel and perpendicular polarized fluorescence signals after image thresholding and background subtraction. (*B*) In situ calibration of the Venus FLARE-Cameleon sensor in SCN neurons. Anisotropy values from regions of SCN slices incubated in solutions with known free $Ca^{2+}$ (0–1 mM) are plotted against $Ca^{2+}$ concentration. Data points (*open black circles*) representing anisotropy measurements from separate imaging regions were fitted with a nonlinear regression (*red line*) overlayed with the mean ± SEM of the anisotropy values from all slices in each $Ca^{2+}$ condition (*closed red squares*). N = 2–4 slices per $Ca^{2+}$ condition with one to three imaging regions per slice. (*C*) Plots of median, 25th and 75th percentile (*boxes*), and minimal and maximal (*whiskers*) anisotropy values at peak and trough time points. (*D*) Box plots of estimated peak and trough $Ca^{2+}$ concentrations calculated from anisotropy values in (*C*). Compared to the trough, peak anisotropy was significantly decreased ($p$ = 0.04) and peak $Ca^{2+}$ concentration was significantly increased ($p$ = 0.02). *$p < 0.05$, unpaired Student's *t*-test. Data points represent measurements from individual SCN slices (one imaging region per slice). N = 39 slices for peak, N = 43 slices for trough.

measurements of $Ca^{2+}$ that are comparable across experimental time points and different SCN slices.

Fluorescent signals from neurons expressing the Venus FLARE-Cameleon biosensor were clearly detectable within the SCN (Fig. 1 *A ii*). Volumetric images of the polarized fluorescence signals were collected from a cubic area of the SCN (Fig. S2). A local threshold, calculated based on the sum from a Gaussian window, was applied to each image to delineate cell signals (above threshold) from background (below threshold). Anisotropies were calculated from integrating the signal from all pixels above the threshold in each image, which were summed across all images (20 images per stack) to provide a single anisotropy distribution per image stack (Fig. 1 *A iii*). Anisotropy histograms were fitted with a Gaussian distribution to calculate a single mean anisotropy value encompassing signals from all cells within the imaging region.

Because the relationship between anisotropy values and $Ca^{2+}$ concentration can be sensitive to variations in temperature and pH (65), the $Ca^{2+}$ concentration and fluorescence anisotropy for Venus FLARE-Cameleon was calibrated in situ from SCN slices incubated in buffered standards of known $Ca^{2+}$ concentration at 35°C (Fig. 1 *B*). For in situ calibration experiments, ionomycin, which permeabilizes the cell membrane to $Ca^{2+}$; rotenone, an ATP inhibitor; and CPA, a SERCA-ATPase inhibitor, were added to the bath solution to limit homeostatic compensation and promote the clamping of intracellular $Ca^{2+}$ concentration (66). We found that the dissociation constant ($K_d$ = 230 nM) and hillslope (n = −1.0) values obtained from this in situ $Ca^{2+}$ calibration curve were similar to those previously reported for this sensor in vitro (42) (Fig. 1 *B*). The maximal (Rmax) and minimal (Rmin) anisotropy values were 0.259 and 0.184 in 0 and 1 mM $Ca^{2+}$, respectively. These results indicated that the Venus FLARE-Cameleon reporter was functionally expressed and responsive to changes in clamped $Ca^{2+}$ concentration.

The in situ calibration values were next used to calculate $Ca^{2+}$ concentrations from images obtained during peak and trough of the circadian cycle. Anisotropy values were calculated from all pixels, in all cells within the imaging region. Each image typically contained between two and five neurons expressing Venus FLARE-Cameleon after image thresholding. Average anisotropies obtained during the peak (0.223 ± 0.002) were significantly lower compared to anisotropies obtained during the trough (0.228 ± 0.002), indicating that more $Ca^{2+}$ was bound to the sensor during the peak of the circadian cycle (Fig. 1 *C*). This corresponded to an estimated $Ca^{2+}$ concentration that was 1.3-fold higher at the peak (218 ± 16 nM; range 53–408 nM) compared to the trough (172 ± 13 nM; range 21–365 nM) (Fig. 1 *D*). These data show that a circadian rhythm in intracellular $Ca^{2+}$ can be detected from SCN neurons with the Venus FLARE-Cameleon sensor, and to our knowledge, provides a new method to track circadian changes in cytosolic $Ca^{2+}$.

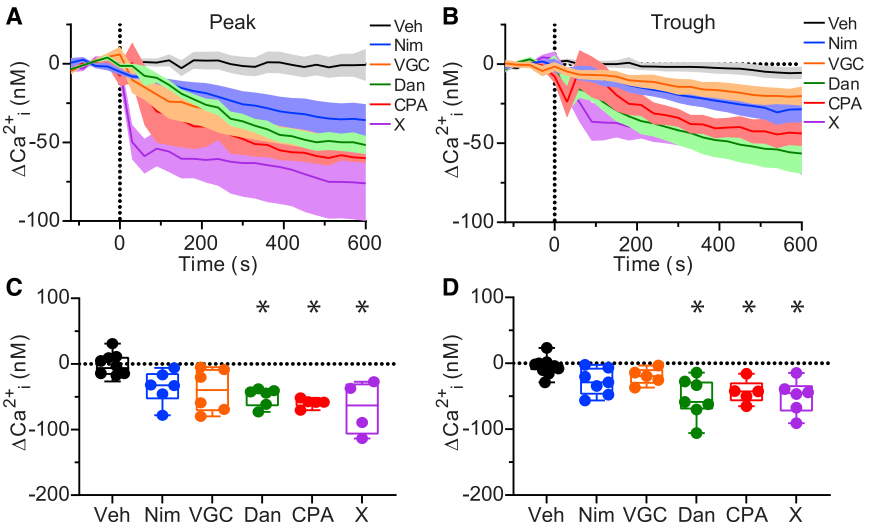


FIGURE 2 Effects of $Ca^{2+}$ channel inhibitors on peak and trough $Ca^{2+}$ concentration. (*A* and *B*) Time course of the change in $Ca^{2+}$ concentration ($\Delta Ca^{2+} = Ca^{2+}$ – average $Ca^{2+}$ from 2-min baseline) before and after the application of vehicle control (Veh) or $Ca^{2+}$ channel inhibitors at the peak (*A*) and trough (*B*). Data are mean ± SEM. (*C* and *D*) Plots of median, 25th and 75th percentile (*boxes*), and minimal and maximal (*whiskers*) changes in $Ca^{2+}$ concentration for individual slices quantified from 9 to 10 min after drugs were applied at the peak (*C*) and trough (*D*). Inhibition of L-type $Ca^{2+}$ channels with nimodipine (Nim, 10 $\mu$M) or inhibition of N/P/Q/R/T-type $Ca^{2+}$ channels with VGC (a mixture of 3 $\mu$M ConoGVIA, 200 nM AgaIVA, 30 $\mu$M nickel, and 1 $\mu$M TTA-P2) did not significantly affect peak or trough $Ca^{2+}$ levels compared to Veh. Inhibition of ryanodine receptors with dantrolene (Dan, 10 $\mu$M), inhibition of SERCA-ATPase with cyclopiazonic acid (CPA, 10 $\mu$M) and combined inhibition of voltage-gated $Ca^{2+}$ channels and ryanodine receptors with a cocktail containing Dan, Nim, and VGC (cocktail X) significantly decreased $Ca^{2+}$ at peak and trough. $*p < 0.05$, one-way ANOVA and Bonferroni post hoc test between drug and vehicle control conditions at peak (Nim, $p = 0.3$; VGC, $p = 0.1$; Dan, $p = 0.02$; CPA, $p = 0.005$; X, $p = 0.003$) and trough (Nim, $p = 0.4$; VGC, $p = 1$; Dan, $p = 0.0004$; CPA, $p = 0.02$; X, $p = 0.002$). Post hoc values were $p > 0.05$ for all other comparisons. Data points represent average measurements from individual SCN slices (one imaging region per slice). N = 4–11 slices per condition.

To define the response of the biosensor to acute changes in $Ca^{2+}$ signaling under these in situ conditions, 50 mM KCl was applied to a subset of SCN slices at the peak and trough. Volumetric images were acquired in 10-s intervals for a 2-min baseline period before KCl was applied to the bath chamber and up to 10 min after KCl application. Consistent with prior studies (67), SCN slices responded to KCl treatment with a transient decrease in anisotropy values (peak: −0.024 ± 0.005; trough: −0.018 ± 0.003), corresponding to a transient increase in neuronal $Ca^{2+}$ (peak: +271 ± 77 nM; trough: +524 ± 212 nM). The maximal KCl-evoked responses were compared with the average baseline anisotropies and $Ca^{2+}$ concentrations at the circadian peak and trough. KCl produced a transient increase in $Ca^{2+}$ levels of 3.66-fold during the peak (baseline: 102 ± 25 nM, KCl: 373 ± 78 nM; n = 5) and 3.52-fold during the trough (baseline: 208 ± 20 nM, KCl: 732 ± 228 nM; n = 8). Thus, Venus FLARE-Cameleon detects changes in $Ca^{2+}$ evoked during maximal $Ca^{2+}$ signaling at both the peak and trough.

Next, to test the contributions of the different voltage-gated and intracellular $Ca^{2+}$ channel subtypes to neuronal $Ca^{2+}$ in the SCN, we measured the effects of $Ca^{2+}$ channel inhibitors on anisotropy (Fig. S3, *A–D*) and estimated $Ca^{2+}$ concentration (Fig. 2, *A–D*) during the peak and trough of the circadian cycle. For $Ca^{2+}$ channel pharmacology experiments, volumetric images were captured in 30-s intervals for 2 min to obtain baseline anisotropy values before drugs or vehicle controls were applied. The effects of each drug were analyzed in slices imaged in 30-s intervals for 2 min of baseline and for 10 min after the application of a drug or vehicle control (Veh, <0.1% DMSO), which were added to the bath solution just before time 0. The average change in anisotropy values (Fig. S3, *A* and *B*) and corresponding change in $Ca^{2+}$ concentrations (Fig. 2, *A* and *B*) relative to the baseline average of each slice were plotted as a function of time. Anisotropy values were stable, with no significant change for the duration of the 2-min baseline recordings in each condition (Fig. S3, *A* and *B*) ($p = 0.3$, two-way repeated-measures ANOVA). As a control for neuronal health after drug treatments, 50 mM KCl was applied to some slices after drug effects were obtained. KCl produced decreases in anisotropy values corresponding to increases in $Ca^{2+}$ concentration that were 2–20 times higher than baseline $Ca^{2+}$ levels (data not shown). The duration and magnitudes of these transient KCl-evoked responses were similar to those observed for slices in control conditions. These KCl responses obtained at the end of the experimental protocol verify that SCN slices are able respond to stimuli after $Ca^{2+}$ channel inhibitors were applied, demonstrating that drug exposure did not affect slice viability.

First, to probe the contributions of voltage-gated channels, we used 10 $\mu$M Nim to target L-type $Ca^{2+}$ channels and a cocktail containing voltage-gated channel inhibitors (VGCs) targeting N-type (3 $\mu$M ConoGVIA), P/Q-type (200 nM AgaIVA), R-type (30 $\mu$M

nickel), and T-type (1 $\mu$M TTA-P2) $Ca^{2+}$ channels. For each slice, paired comparisons were made between baseline, and the $Ca^{2+}$ concentration averaged from 9 to 10 min after drugs were applied (Fig. S3, *C–F*). Application of vehicle control (Veh, <0.1% DMSO) during the peak or the trough did not significantly affect anisotropy values (Fig. S3, *C* and *D*) or $Ca^{2+}$ concentrations (Fig. S3, *E* and *F*) compared to baseline. In contrast, voltage-gated $Ca^{2+}$ channel inhibitors produced an increase in anisotropy values (Fig. S3, *C* and *D*), which corresponded to a reduction in the $Ca^{2+}$ levels in paired comparison to baseline values (Fig. S3, *E* and *F*). These data implicate voltage-gated $Ca^{2+}$ channels as contributors to the cytosolic $Ca^{2+}$ levels during the peak and the trough.

We then tested the contributions of intracellular $Ca^{2+}$ channels. Previous studies have shown that inhibiting RyR-mediated $Ca^{2+}$ release from the ER produced a decrease in cytosolic $Ca^{2+}$ levels (24,41), but the effect of inhibiting intracellular $Ca^{2+}$ channels at both the peak and trough has not been systematically tested. 10 $\mu$M Dan was used to inhibit RyRs, and 10 $\mu$M CPA was used to target the SERCA-ATPase, which inhibits refilling of ER $Ca^{2+}$ stores. CPA produces ER store depletion and subsequent inhibition of both RyR-and $IP_3R$-mediated $Ca^{2+}$ release (68). In a third condition, a cocktail (X) containing a combination of the VGCs, plus nimodipine and dantrolene to collectively inhibit RyRs along with the voltage-gated channels, was applied. Application of cocktail X thus inhibits other channels without blocking $IP_3Rs$. We found that each inhibitor of intracellular $Ca^{2+}$ channels produced a significant decrease in $Ca^{2+}$ levels at the peak and the trough in a paired comparison to the baseline values for each slice (Fig. S3, *E* and *F*). These results suggest that intracellular channels also contribute to cytosolic $Ca^{2+}$ levels at both the peak and the trough.

Determination of the relative contributions for VGCCs and intracellular $Ca^{2+}$ channels across experiments requires accounting for the variation in baseline $Ca^{2+}$ levels in each SCN slice. To make this comparison, the average $Ca^{2+}$ concentration from 2 min of baseline was subtracted from the average $Ca^{2+}$ levels 9–10 min after drug application to obtain the change in $Ca^{2+}$ ($\Delta Ca^{2+}$) within each slice. First, we focused on the $\Delta Ca^{2+}$ produced by each drug during the peak (Fig. 2, *A* and *C*). Starting with the vehicle controls, the $\Delta Ca^{2+}$ was negligible at $-2 \pm 7$ nM ($-2 \pm 5$% change) (Fig. 2 *C*). The voltage-gated channel inhibitors produced a $\Delta Ca^{2+}$ of $-35 \pm 10$ nM ($-20 \pm 4$% change) (Nim) and $-40 \pm 13$ nM ($-19 \pm 4$%) (VGC); however, these decreases were not statistically different than the vehicle control. This may be partly explained by the variability in the $\Delta Ca^{2+}$ responses of individual slices, which ranged from $-6$ to $-78$ nM (Nim) and $-5$ to $-79$ nM (VGC) (Fig. 2 *C*). In contrast, intracellular $Ca^{2+}$ channel inhibitors produced decreases in peak $Ca^{2+}$ that were significantly larger than the vehicle controls. Dantrolene produced the largest decrease, with a $\Delta Ca^{2+}$ of $-50 \pm 6$ nM ($-36 \pm 6$% change). Similarly, the SERCA inhibitor CPA produced a $\Delta Ca^{2+}$ of $-59 \pm 3$ nM ($-24 \pm 5$%), and the mixture of VGC inhibitors along with nimodipine and dantrolene added together (X cocktail) produced a $\Delta Ca^{2+}$ of $-67 \pm 21$ nM ($-22 \pm 6$%) (Fig. 2 *C*). The responses of individual slices to intracellular $Ca^{2+}$ channel inhibitors were less variable, with the range of $-38$ to $-73$ nM for dantrolene, $-52$ to $-71$ nM for CPA, and $-27$ to $-114$ nM for cocktail X. Taken together, these results suggest that peak intracellular $Ca^{2+}$ is predominantly set by RyR channel contribution.

These inhibitors had similar effects on $Ca^{2+}$ during the trough of the circadian cycle (Fig. 2 *D*). Vehicle control had little effect, $-5 \pm 4$ nM ($-7 \pm 5$% change). Nimodipine decreased $Ca^{2+}$ by $-28 \pm 8$ nM ($-17 \pm 5$%) overall with a range of responses between $-2$ and $-56$ nM. Similarly, VGC decreased $Ca^{2+}$ by $-20 \pm 6$ nM ($-16 \pm 3$%), with changes in $Ca^{2+}$ ranging from $-3.5$ to $-37$ nM. The decreases in trough $Ca^{2+}$ were significantly larger for dantrolene, $-53 \pm 12$ nM ($-28 \pm 6$%); CPA, $-43 \pm 8$ nM ($-32 \pm 7$%); and cocktail X, $-51 \pm 11$ nM ($-23 \pm 5$%). These overall decreases were accompanied by a larger range in $\Delta Ca^{2+}$ responses, which were $-14$ to $-106$ nM for dantrolene, $-16$ to $-65$ nM for CPA, and $-15$ to $-91$ nM for cocktail X (Fig. 2 *D*). Thus, at both peak and trough time points, the largest decrease in $Ca^{2+}$ was produced by dantrolene. In contrast, the contribution of voltage-gated channels appears less significant and in some cases has more variability.

## DISCUSSION

A primary advance of this study, to our knowledge, is the novel application of Venus-cp172Venus FLARE-Cameleon sensor (42) with piSPIM (59) to measure biologically controlled changes in $Ca^{2+}$ in live organotypic brain tissue. The Venus FLARE-Cameleon $Ca^{2+}$ sensor captured the collective signal in basal cytosolic $Ca^{2+}$ averaged from multiple neurons within a region of the SCN without integrating shorter-timescale $Ca^{2+}$ signals, such as action-potential-evoked $Ca^{2+}$ transients. Using these techniques, $Ca^{2+}$ concentrations ($218 \pm 16$ and $172 \pm 13$ nM, respectively) were refined over prior absolute and relativistic estimates (27). These SCN concentrations are consistent with basal $Ca^{2+}$ levels typically measured in other neuronal cell types, ~40–190 nM (69), and the values reported using other ratiometric $Ca^{2+}$ sensors in the SCN. In prior studies, peak values ranged between 50 and 440 nM, and trough values ranged between 50 and 150 nM (27). However, prior

measurements using Fura-2 (13,29,41,70) or genetically encoded sensors such as Yellow Cameleons (24,25,33,71) did not utilize in situ calibration of the $Ca^{2+}$ sensor. These measurements relied on cell-free in vitro calibrations, which does not account for factors in the intracellular environment that could affect the $Ca^{2+}$ estimates (65). In this study, $Ca^{2+}$ measurements were obtained using a $Ca^{2+}$ sensor that was calibrated in SCN slices under the same experimental conditions at baseline and across different $Ca^{2+}$ inhibitor experiments. Venus FLARE-Cameleon also has the added advantage of only occupying a single-color channel, which will allow imaging of multiple biosensors expressed in the same neuron. Thus, this study provides a foundation for future experiments to investigate the cross talk between $Ca^{2+}$ and other cellular signaling components toward piecing together how the ensemble circadian clock mechanism functions at a cellular level.

A central feature of SCN neurons is that they express different properties depending on the time of the circadian cycle. SCN neurons exhibit a state of increased excitability and increased activation of voltage-gated $Ca^{2+}$ channels during the day (peak of the cycle) and a state of decreased excitability at night (trough of the cycle), during which voltage-gated $Ca^{2+}$ channel activity is reduced (3,4,38,72). Yet, it has remained unclear whether this daily increase in voltage-gated $Ca^{2+}$ channel activity is involved in maintaining the circadian pattern in cytosolic $Ca^{2+}$, which is also highest during the circadian peak. Furthermore, no single study has directly compared the contributions for these different $Ca^{2+}$ channel types to cytosolic $Ca^{2+}$ levels at both peak and trough of the circadian cycle in intact SCN slices. Prior studies measured the effects of inhibitors on $Ca^{2+}$ levels only at a single time point or employed only a single $Ca^{2+}$ channel inhibitor (24,28,30,41,67). As a result of these methodological discrepancies, the relative contributions of the $Ca^{2+}$ channel subtypes at both times of the circadian cycle have been less than fully conclusive. For example, in studies measuring the $Ca^{2+}$ rhythms from the whole SCN, inhibition of L-type voltage-gated $Ca^{2+}$ channels with nimodipine reduced the magnitude of the day-night difference in $Ca^{2+}$ levels (28,30). However, other studies found no effect of nimodipine on $Ca^{2+}$ levels (24,73).

With direct comparison of the relative contributions for each $Ca^{2+}$ source under equivalent experimental conditions, the results in this study support the current view that intracellular RyR $Ca^{2+}$ channels are major contributors to the $Ca^{2+}$ levels during both the peak and trough of the circadian cycle. Because the combined inhibition of voltage-gated channels and RyRs did not significantly decrease $Ca^{2+}$ levels further compared to inhibiting RyRs alone, it suggests that RyR inhibition produced the majority of the decrease in $Ca^{2+}$. Inhibiting the SERCA-ATPase, which prevents $Ca^{2+}$ reuptake into the ER and leads to a depletion of ER stores (68), also did not produce a decrease in $Ca^{2+}$ that was larger in magnitude than the decrease observed when inhibiting RyRs alone. This further suggests that $IP_3Rs$, which also can mediate ER $Ca^{2+}$ release, may have a lesser contribution to cytosolic $Ca^{2+}$ compared to RyRs, although this was not tested directly because of a lack of selective $IP_3R$ inhibitors (74). This study corroborates prior reports of decreased peak $Ca^{2+}$ with RyR inhibition (6,24,41). RyR2 messenger RNA (mRNA) and protein also exhibit a daytime peak in expression (38,75). However, it is unlikely that an expression-based mechanism would fully account for the circadian oscillation in $Ca^{2+}$ levels, as RyR activity is regulated by increases in intracellular $Ca^{2+}$ (27). Although it remains to be determined whether other VGCCs contribute to $Ca^{2+}$-induced $Ca^{2+}$ release in SCN neurons, the lesser effect of inhibiting these channels suggests they do not serve as the primary sensors for RyR-mediated $Ca^{2+}$ release. Other sources of calcium in the SCN not tested here include ionotropic glutamate receptors, including *N*-methyl-D-aspartate receptors and $\alpha$-amino-3-hydroxy-5-methyl-4-isoxazolepropionic acid receptors (20). $Ca^{2+}$ homeostasis is also maintained by the activity of $Na^{+}$-$Ca^{2+}$ exchanger types 1 and 2 (NCX1 and NCX2) (38,75) and endoplasmic reticulum $Ca^{2+}$-ATPases (SERCA) (70), which mediate $Ca^{2+}$ efflux or uptake into ER stores, whereas uptake of $Ca^{2+}$ via mitochondrial NCX (76) and $Ca^{2+}$-binding proteins buffer cytosolic $Ca^{2+}$ (77–79). There is also potential involvement of store-operated $Ca^{2+}$ entry channels (80). Another mechanism that could be involved in regulating basal $Ca^{2+}$ over the circadian cycle, in conjunction with the activity of ion channels, is $Ca^{2+}$ buffering by $Ca^{2+}$-binding proteins (69), which can alter basal $Ca^{2+}$ as well as influence the amplitude and decay of stimulus-evoked $Ca^{2+}$ transients (81,82), leading to changes in firing properties of neurons (69). SCN neurons express calbindin $D_{28K}$ and calretinin (77,83). In SCN, levels of cytosolic calbindin protein have been observed to change over the course of the circadian cycle (77). This evidence suggests that these $Ca^{2+}$ binding proteins can be regulated based on the daily requirements of SCN neurons and could play an important role in circadian $Ca^{2+}$ rhythms. Further studies will be required to investigate their contributions.

This study did not focus on defining the $Ca^{2+}$ regulatory mechanisms by subregion, but the phase of the $Ca^{2+}$ rhythm exhibits regional differences. It has not yet been addressed whether the $Ca^{2+}$ channels themselves differ by subregion as the basis. The shell region of the SCN, defined by expression of the neuropeptide arginine vasopressin, exhibits a rhythmic

$Ca^{2+}$ peak 3–5 h before the core, defined by vasoactive intestinal polypeptide (VIP) expression (28,30,33). Shell $Ca^{2+}$ rhythms also had higher amplitudes. These regional phase and amplitude differences could contribute to the wide variation in $Ca^{2+}$ values observed in the baseline measurements of this study, as the ROIs were located in the center of the SCN and undefined with respect to the core and shell boundaries. In addition, sequential application of inhibitors could reveal whether the relative contribution of $Ca^{2+}$ channel types differs between subregions. However, in previous studies, at least one inhibitor (nimodipine) failed to show a regional difference in its effects on $Ca^{2+}$ levels (30), leaving open the question of which $Ca^{2+}$ channels produce these phase differences. Regional differences are also present in *Drosophila* clock neurons (84,85), in which basal $Ca^{2+}$ levels were sensitive to RNA interference (RNAi) knockdown of intracellular $Ca^{2+}$ release via $IP_3Rs$ or SERCA, but only one set of neurons was sensitive to VGCC knockdown (86).

## SUPPORTING MATERIAL

Supplemental information can be found online at https://doi.org/10.1016/j.bpr.2021.100005.

## AUTHOR CONTRIBUTIONS

A.E.P. and A.L.M. designed the experiments and wrote the manuscript. A.E.P. performed the experiments and analyzed the data, V.P.R. wrote the computer code and assisted with image analysis, and M.A.R. designed and built the piSPIM microscope.

## ACKNOWLEDGMENTS

We thank Joe Mauban, the University of Maryland Confocal Core Facility, and members of the Blanpied lab for assistance with confocal imaging. We thank members of the Meredith lab for comments on the manuscript.

This work was supported by grants from NHLBI R01-HL102758 (A.L.M.), the Training Program in Integrative Membrane Biology NHLBI T32-GM008181 (A.L.M., V.P.R., and A.E.P.), NIMH R01-MH111527 (A.L.M. and M.A.R.), NIH Fellowship Grant F30DK124986 (V.P.R.), NIDDK R01DK077140 (M.A.R.), and NHLBI R01HL122827 (M.A.R.).

## DECLARATION OF INTERESTS

The authors declare no competing interests.

## SUPPORTING CITATIONS

References (87–89) appear in the Supporting material.

## REFERENCES

1. Lundkvist, G. B., Y. Kwak, …, G. D. Block. 2005. A calcium flux is required for circadian rhythm generation in mammalian pacemaker neurons. *J. Neurosci.* 25:7682–7686.
2. Nahm, S. S., Y. Z. Farnell, …, D. J. Earnest. 2005. Circadian regulation and function of voltage-dependent calcium channels in the suprachiasmatic nucleus. *J. Neurosci.* 25:9304–9308.
3. McNally, B. A., A. E. Plante, and A. L. Meredith. 2020. Diurnal properties of voltage-gated $Ca^{2+}$ currents in suprachiasmatic nucleus and roles in action potential firing. *J. Physiol.* 598:1775–1790.
4. Jackson, A. C., G. L. Yao, and B. P. Bean. 2004. Mechanism of spontaneous firing in dorsomedial suprachiasmatic nucleus neurons. *J. Neurosci.* 24:7985–7998.
5. Pennartz, C. M., M. T. de Jeu, …, A. M. Geurtsen. 2002. Diurnal modulation of pacemaker potentials and calcium current in the mammalian circadian clock. *Nature.* 416:286–290.
6. Aguilar-Roblero, R., C. Mercado, …, M. Díaz-Muñoz. 2007. Ryanodine receptor $Ca^{2+}$-release channels are an output pathway for the circadian clock in the rat suprachiasmatic nuclei. *Eur. J. Neurosci.* 26:575–582.
7. Moore, R. Y., and V. B. Eichler. 1972. Loss of a circadian adrenal corticosterone rhythm following suprachiasmatic lesions in the rat. *Brain Res.* 42:201–206.
8. Silver, R., J. LeSauter, …, M. N. Lehman. 1996. A diffusible coupling signal from the transplanted suprachiasmatic nucleus controlling circadian locomotor rhythms. *Nature.* 382:810–813.
9. Meyer-Bernstein, E. L., A. E. Jetton, …, E. L. Bittman. 1999. Effects of suprachiasmatic transplants on circadian rhythms of neuroendocrine function in golden hamsters. *Endocrinology.* 140:207–218.
10. Schwartz, W. J., R. A. Gross, and M. T. Morton. 1987. The suprachiasmatic nuclei contain a tetrodotoxin-resistant circadian pacemaker. *Proc. Natl. Acad. Sci. USA.* 84:1694–1698.
11. Ralph, M. R., R. G. Foster, …, M. Menaker. 1990. Transplanted suprachiasmatic nucleus determines circadian period. *Science.* 247:975–978.
12. Stephan, F. K., and I. Zucker. 1972. Circadian rhythms in drinking behavior and locomotor activity of rats are eliminated by hypothalamic lesions. *Proc. Natl. Acad. Sci. USA.* 69:1583–1586.
13. Irwin, R. P., and C. N. Allen. 2007. Calcium response to retinohypothalamic tract synaptic transmission in suprachiasmatic nucleus neurons. *J. Neurosci.* 27:11748–11757.
14. Kim, D. Y., H. J. Choi, …, Y. I. Kim. 2005. Voltage-gated calcium channels play crucial roles in the glutamate-induced phase shifts of the rat suprachiasmatic circadian clock. *Eur. J. Neurosci.* 21:1215–1222.
15. Hirota, T., and Y. Fukada. 2004. Resetting mechanism of central and peripheral circadian clocks in mammals. *Zool. Sci.* 21:359–368.
16. Meijer, J. H., and W. J. Schwartz. 2003. In search of the pathways for light-induced pacemaker resetting in the suprachiasmatic nucleus. *J. Biol. Rhythms.* 18:235–249.
17. van den Pol, A. N., S. M. Finkbeiner, and A. H. Cornell-Bell. 1992. Calcium excitability and oscillations in suprachiasmatic nucleus neurons and glia in vitro. *J. Neurosci.* 12:2648–2664.
18. van den Pol, A. N. 1993. Glutamate and GABA presence and action in the suprachiasmatic nucleus. *J. Biol. Rhythms.* 8 (*Suppl*):S11–S15.
19. Jones, J. R., T. Simon, …, E. D. Herzog. 2018. SCN VIP neurons are essential for normal light-mediated resetting of the circadian system. *J. Neurosci.* 38:7986–7995.
20. Tominaga, K., M. E. Geusz, …, S. T. Inouye. 1994. Calcium imaging in organotypic cultures of the rat suprachiasmatic nucleus. *Neuroreport.* 5:1901–1905.

21. Tischkau, S. A., J. W. Mitchell, …, M. U. Gillette. 2003. $Ca^{2+}$/cAMP response element-binding protein (CREB)-dependent activation of Per1 is required for light-induced signaling in the suprachiasmatic nucleus circadian clock. *J. Biol. Chem.* 278:718–723.
22. Ding, J. M., G. F. Buchanan, …, M. U. Gillette. 1998. A neuronal ryanodine receptor mediates light-induced phase delays of the circadian clock. *Nature.* 394:381–384.
23. Akiyama, M., Y. Kouzu, …, S. Shibata. 1999. Inhibition of light- or glutamate-induced mPer1 expression represses the phase shifts into the mouse circadian locomotor and suprachiasmatic firing rhythms. *J. Neurosci.* 19:1115–1121.
24. Ikeda, M., T. Sugiyama, …, C. N. Allen. 2003. Circadian dynamics of cytosolic and nuclear $Ca^{2+}$ in single suprachiasmatic nucleus neurons. *Neuron.* 38:253–263.
25. Enoki, R., D. Ono, …, K. Honma. 2012. Single-cell resolution fluorescence imaging of circadian rhythms detected with a Nipkow spinning disk confocal system. *J. Neurosci. Methods.* 207:72–79.
26. Brancaccio, M., A. P. Patton, …, M. H. Hastings. 2017. Astrocytes control circadian timekeeping in the suprachiasmatic nucleus via glutamatergic signaling. *Neuron.* 93:1420–1435.e5.
27. Harvey, J. R. M., A. E. Plante, and A. L. Meredith. 2020. Ion channels controlling circadian rhythms in suprachiasmatic nucleus excitability. *Physiol. Rev.* 100:1415–1454.
28. Noguchi, T., T. L. Leise, …, D. K. Welsh. 2017. Calcium circadian rhythmicity in the suprachiasmatic nucleus: cell autonomy and network modulation. *eNeuro.* 4:ENEURO.0160-17.2017.
29. Colwell, C. S. 2000. Circadian modulation of calcium levels in cells in the suprachiasmatic nucleus. *Eur. J. Neurosci.* 12:571–576.
30. Enoki, R., Y. Oda, …, K. I. Honma. 2017. Synchronous circadian voltage rhythms with asynchronous calcium rhythms in the suprachiasmatic nucleus. *Proc. Natl. Acad. Sci. USA.* 114:E2476–E2485.
31. Ono, D., S. Honma, …, K. I. Honma. 2017. Dissociation of *Per1* and *Bmal1* circadian rhythms in the suprachiasmatic nucleus in parallel with behavioral outputs. *Proc. Natl. Acad. Sci. USA.* 114:E3699–E3708.
32. Patton, A. P., M. D. Edwards, …, M. H. Hastings. 2020. The VIP-VPAC2 neuropeptidergic axis is a cellular pacemaking hub of the suprachiasmatic nucleus circadian circuit. *Nat. Commun.* 11:3394.
33. Enoki, R., S. Kuroda, …, K. Honma. 2012. Topological specificity and hierarchical network of the circadian calcium rhythm in the suprachiasmatic nucleus. *Proc. Natl. Acad. Sci. USA.* 109:21498–21503.
34. Hong, J. H., B. Jeong, …, K. J. Lee. 2012. Circadian waves of cytosolic calcium concentration and long-range network connections in rat suprachiasmatic nucleus. *Eur. J. Neurosci.* 35:1417–1425.
35. Brancaccio, M., E. S. Maywood, …, M. H. Hastings. 2013. A Gq-$Ca^{2+}$ axis controls circuit-level encoding of circadian time in the suprachiasmatic nucleus. *Neuron.* 78:714–728.
36. Ding, J. M., D. Chen, …, M. U. Gillette. 1994. Resetting the biological clock: mediation of nocturnal circadian shifts by glutamate and NO. *Science.* 266:1713–1717.
37. Schurov, I. L., S. McNulty, …, M. H. Hastings. 1999. Glutamatergic induction of CREB phosphorylation and Fos expression in primary cultures of the suprachiasmatic hypothalamus in vitro is mediated by co-ordinate activity of NMDA and non-NMDA receptors. *J. Neuroendocrinol.* 11:43–51.
38. Díaz-Muñoz, M., M. A. Dent, …, R. Aguilar-Roblero. 1999. Circadian modulation of the ryanodine receptor type 2 in the SCN of rodents. *Neuroreport.* 10:481–486.
39. Hamada, T., S. Y. Liou, …, N. Ishida. 1999. The role of inositol trisphosphate-induced $Ca^{2+}$ release from IP3-receptor in the rat suprachiasmatic nucleus on circadian entrainment mechanism. *Neurosci. Lett.* 263:125–128.
40. Cheng, P. C., Y. C. Wang, …, R. C. Huang. 2018. Differential regulation of nimodipine-sensitive and -insensitive $Ca^{2+}$ influx by the $Na^{+}/Ca^{2+}$ exchanger and mitochondria in the rat suprachiasmatic nucleus neurons. *J. Biomed. Sci.* 25:44.
41. Aguilar-Roblero, R., D. Quinto, …, G. Lundkvist. 2016. Ryanodine-sensitive intracellular $Ca^{2+}$ channels are involved in the output from the SCN circadian clock. *Eur. J. Neurosci.* 44:2504–2514.
42. Ross, B. L., B. Tenner, …, J. Zhang. 2018. Single-color, ratiometric biosensors for detecting signaling activities in live cells. *eLife.* 7:e35458.
43. Montgomery, J. R., J. P. Whitt, …, A. L. Meredith. 2013. Mis-expression of the BK $K^{(+)}$ channel disrupts suprachiasmatic nucleus circuit rhythmicity and alters clock-controlled behavior. *Am. J. Physiol. Cell Physiol.* 304:C299–C311.
44. Kügler, S., E. Kilic, and M. Bähr. 2003. Human synapsin 1 gene promoter confers highly neuron-specific long-term transgene expression from an adenoviral vector in the adult rat brain depending on the transduced area. *Gene Ther.* 10:337–347.
45. Whitt, J. P., J. R. Montgomery, and A. L. Meredith. 2016. BK channel inactivation gates daytime excitability in the circadian clock. *Nat. Commun.* 7:10837.
46. Kumar, A., Y. Wu, …, H. Shroff. 2014. Dual-view plane illumination microscopy for rapid and spatially isotropic imaging. *Nat. Protoc.* 9:2555–2573.
47. Kumar, A., R. Christensen, …, H. Shroff. 2016. Using stage- and slit-scanning to improve contrast and optical sectioning in dual-view inverted light sheet microscopy (diSPIM). *Biol. Bull.* 231:26–39.
48. Wu, Y., P. Wawrzusin, …, H. Shroff. 2013. Spatially isotropic four-dimensional imaging with dual-view plane illumination microscopy. *Nat. Biotechnol.* 31:1032–1038.
49. Edelstein, A. D., M. A. Tsuchida, …, N. Stuurman. 2014. Advanced methods of microscope control using μManager software. *J. Biol. Methods.* 1:e10.
50. Ross, B., S. H. Wong, …, M. A. Rizzo. 2019. Triple fluorescence anisotropy reporter imaging in living cells. *Bio Protoc.* 9:ew3226.
51. Gomez, G. 2000. Local smoothness in terms of variance: the adaptive Gaussian filter. *In* Proceedings of the BMVC 2000. M. Mirmehdi and B. Thomas, eds. BMVA Press, pp. 815–824.
52. Sezgin, M., and B. Sankur. 2004. Survey over image thresholding techniques and quantitative performance evaluation. *J. Electron. Imaging.* 13:146–166.
53. Rizzo, M. A., and D. W. Piston. 2005. High-contrast imaging of fluorescent protein FRET by fluorescence polarization microscopy. *Biophys. J.* 88:L14–L16.
54. McGuigan, J. A., D. Lüthi, and A. Buri. 1991. Calcium buffer solutions and how to make them: a do it yourself guide. *Can. J. Physiol. Pharmacol.* 69:1733–1749.
55. Miyawaki, A., J. Llopis, …, R. Y. Tsien. 1997. Fluorescent indicators for $Ca^{2+}$ based on green fluorescent proteins and calmodulin. *Nature.* 388:882–887.
56. Miyawaki, A., O. Griesbeck, …, R. Y. Tsien. 1999. Dynamic and quantitative $Ca^{2+}$ measurements using improved cameleons. *Proc. Natl. Acad. Sci. USA.* 96:2135–2140.
57. Heim, N., O. Garaschuk, …, O. Griesbeck. 2007. Improved calcium imaging in transgenic mice expressing a troponin C-based biosensor. *Nat. Methods.* 4:127–129.
58. Nagai, T., S. Yamada, …, A. Miyawaki. 2004. Expanded dynamic range of fluorescent indicators for $Ca^{(2+)}$ by circularly permuted yellow fluorescent proteins. *Proc. Natl. Acad. Sci. USA.* 101:10554–10559.
59. Markwardt, M. L., N. E. Snell, …, M. A. Rizzo. 2018. A genetically encoded biosensor strategy for quantifying non-muscle myosin II phosphorylation dynamics in living cells and organisms. *Cell Rep.* 24:1060–1070.e4.

60. Jares-Erijman, E. A., and T. M. Jovin. 2003. FRET imaging. *Nat. Biotechnol.* 21:1387–1395.

61. Mattheyses, A. L., A. D. Hoppe, and D. Axelrod. 2004. Polarized fluorescence resonance energy transfer microscopy. *Biophys. J.* 87:2787–2797.

62. Tian, L., S. A. Hires, …, L. L. Looger. 2009. Imaging neural activity in worms, flies and mice with improved GCaMP calcium indicators. *Nat. Methods.* 6:875–881.

63. Rizzo, M. A., G. Springer, …, D. W. Piston. 2006. Optimization of pairings and detection conditions for measurement of FRET between cyan and yellow fluorescent proteins. *Microsc. Microanal.* 12:238–254.

64. Rizzo, M. A., and D. W. Piston. 2005. High-contrast imaging of fluorescent protein FRET by fluorescence polarization microscopy. *Biophys J.* 88 (2):14–16.

65. Glaum, S. R., S. Alford, …, N. T. Slater. 1994. Whole-cell patch recording with simultaneous measurement of intracellular calcium concentration in mammalian brain slices in vitro. *In* Methods in Neurosciences. T. Narahashi, ed. Elsevier, pp. 340–358.

66. Thomas, D., S. C. Tovey, …, P. Lipp. 2000. A comparison of fluorescent $Ca^{2+}$ indicator properties and their use in measuring elementary and global $Ca^{2+}$ signals. *Cell Calcium.* 28:213–223.

67. Cheng, R. C., P. C. Cheng, …, R. C. Huang. 2019. Role of intracellular $Na^{+}$ in the regulation of $[Ca^{2+}]_i$ in the rat suprachiasmatic nucleus neurons. *Int. J. Mol. Sci.* 20:E4868.

68. Garaschuk, O., Y. Yaari, and A. Konnerth. 1997. Release and sequestration of calcium by ryanodine-sensitive stores in rat hippocampal neurones. *J. Physiol.* 502:13–30.

69. Schwaller, B. 2010. Cytosolic $Ca^{2+}$ buffers. *Cold Spring Harb. Perspect. Biol.* 2:a004051.

70. Burkeen, J. F., A. D. Womac, …, M. J. Zoran. 2011. Mitochondrial calcium signaling mediates rhythmic extracellular ATP accumulation in suprachiasmatic nucleus astrocytes. *J. Neurosci.* 31:8432–8440.

71. Enoki, R., D. Ono, …, K. I. Honma. 2017. Dual origins of the intracellular circadian calcium rhythm in the suprachiasmatic nucleus. *Sci. Rep.* 7:41733.

72. de Jeu, M., M. Hermes, and C. Pennartz. 1998. Circadian modulation of membrane properties in slices of rat suprachiasmatic nucleus. *Neuroreport.* 9:3725–3729.

73. Ikeda, M., and M. Ikeda. 2014. Bmal1 is an essential regulator for circadian cytosolic $Ca^{2+}$ rhythms in suprachiasmatic nucleus neurons. *J. Neurosci.* 34:12029–12038.

74. Bootman, M. D., T. J. Collins, …, C. M. Peppiatt. 2002. 2-aminoethoxydiphenyl borate (2-APB) is a reliable blocker of store-operated $Ca^{2+}$ entry but an inconsistent inhibitor of InsP3-induced $Ca^{2+}$ release. *FASEB J.* 16:1145–1150.

75. Aguilar-Roblero, R., M. Díaz-Muñoz, …, S. Michel. 2015. Intracellular calcium as a clock output from SCN neurons. *In* Mechanisms of Circadian Systems in Animals and Their Clinical Relevance. R. Aguilar-Roblero, M. Díaz-Muñoz, and M. Fanjul-Moles, eds. Springer, pp. 115–132.

76. Wang, Y. C., Y. S. Chen, …, R. C. Huang. 2015. Role of $Na^{+}/Ca^{2+}$ exchanger in $Ca^{2+}$ homeostasis in rat suprachiasmatic nucleus neurons. *J. Neurophysiol.* 113:2114–2126.

77. Hamada, T., J. LeSauter, …, R. Silver. 2003. Calbindin influences response to photic input in suprachiasmatic nucleus. *J. Neurosci.* 23:8820–8826.

78. Carafoli, E. 2002. Calcium signaling: a tale for all seasons. *Proc. Natl. Acad. Sci. USA.* 99:1115–1122.

79. Bryant, D. N., J. LeSauter, …, M. T. Romero. 2000. Retinal innervation of calbindin-D28K cells in the hamster suprachiasmatic nucleus: ultrastructural characterization. *J. Biol. Rhythms.* 15:103–111.

80. Kononenko, N. I., I. Medina, and F. E. Dudek. 2004. Persistent subthreshold voltage-dependent cation single channels in suprachiasmatic nucleus neurons. *Neuroscience.* 129:85–92.

81. Chard, P. S., D. Bleakman, …, R. J. Miller. 1993. Calcium buffering properties of calbindin D28k and parvalbumin in rat sensory neurones. *J. Physiol.* 472:341–357.

82. Matthews, E. A., and D. Dietrich. 2015. Buffer mobility and the regulation of neuronal calcium domains. *Front. Cell. Neurosci.* 9:48.

83. Silver, R., M. T. Romero, …, J. LeSauter. 1996. Calbindin-D28K cells in the hamster SCN express light-induced Fos. *Neuroreport.* 7:1224–1228.

84. Liang, X., T. E. Holy, and P. H. Taghert. 2016. Synchronous Drosophila circadian pacemakers display nonsynchronous $Ca^{2+}$ rhythms in vivo. *Science.* 351:976–981.

85. Liang, X., T. E. Holy, and P. H. Taghert. 2017. A series of suppressive signals within the Drosophila circadian neural circuit generates sequential daily outputs. *Neuron.* 94:1173–1189.e4.

86. Liang, X., T. E. Holy, and P. H. Taghert. 2021. Circadian pacemaker neurons display co-phasic rhythms in basal calcium level and in fast calcium fluctuations. *bioRxiv* https://doi.org/10.1101/2021.05.03.442479.

87. Schindelin, J., I. Arganda-Carreras, …, A. Cardona. 2012. Fiji: an open-source platform for biological-image analysis. *Nat. Methods.* 9:676–682.

88. Thévenaz, P., U. E. Ruttimann, and M. Unser. 1998. A pyramid approach to subpixel registration based on intensity. *IEEE Trans. Image Process.* 7:27–41.

89. Chen, T. W., T. J. Wardill, …, D. S. Kim. 2013. Ultrasensitive fluorescent proteins for imaging neuronal activity. *Nature.* 499:295–300.

**Biophysical Reports, Volume *1***

## Supplemental information

# Comparative $Ca^{2+}$ channel contributions to intracellular $Ca^{2+}$ levels in the circadian clock

**Amber E. Plante, Vishnu P. Rao, Megan A. Rizzo, and Andrea L. Meredith**

# Supporting Material

## Comparative $Ca^{2+}$ channel contributions to intracellular $Ca^{2+}$ levels in the circadian clock

Amber E. Plante, Vishnu P. Rao, Megan A. Rizzo and Andrea L. Meredith
Dept. of Physiology, University of Maryland School of Medicine, Baltimore, MD, 21201

### SUPPLEMENTAL MATERIALS AND METHODS

*Organotypic slices cultured on MEAs*

For confocal imaging experiments, freshly dissected SCN slices were plated onto MEA probes (Alpha MED Scientific Inc., Osaka, Japan; MED-P210A) that were pre-treated overnight with polyethyleneimine (0.1% w/v, Millipore Sigma; P3143) in borate buffer solution (25 mM, pH 8.0, Millipore Sigma; S9640) and rinsed with sterile water as per the manufacturer's instructions (Alpha MED Scientific Inc.; available at https://www.med64.com/documentation). MEA probe dishes were placed in 100 mm petri dishes, surrounded by ~7 mL sterile water in a humidified incubator (37°C, 5% $CO_2$). Slices were maintained in 200-300 µL culture medium. Medium (50%) was exchanged every ~48 hours for the first 8 days in culture. On culture day 3, SCN slices cultured on MEAs were transduced with 0.3 µL of AAV1 containing GCaMP6f cDNA expressed under the hSyn1 promoter (AAV1.Syn1.GCaMP6f.SV40.WPRE; stock $1x10^{13}$ vg/mL, Addgene, #100837-AAV1) (Figure S1A) and cultured for an additional 14-21 days. On culture day 10 and thereafter, MEA dishes were sealed with vacuum grease and glass coverslips and 100% of the medium was exchanged every 72 hours.

*MEA activity recording and analysis*

Circadian rhythms in action potential frequency were recorded from SCN slices on MEAs between culture days 14–21 using a MED64-Plex8 system (Alpha MED Scientific Inc.) as described previously (1). Spontaneous action potential activity was acquired for 3 full circadian cycles (72 hours) in 5-second sweeps every 5 min, with setting low cut frequency 100 Hz and high cut frequency 10,000 Hz (Mobius vWin7, Alpha MED Scientific Inc.). Firing frequency was determined from each 5-second sweep using threshold-based counting of downward-spiking action potentials at ~1.5× the level of baseline noise (typically between −10 and −25 µV). Raw action potential frequencies from 3 circadian cycles from all electrodes visually identified to be within the boundaries of the SCN were plotted in Microsoft Excel. Activity recordings were considered rhythmic if the raw action potential frequency peaked once every ~24 hours with a maximum frequency ≥ 3 Hz compared to the minimum (trough) frequency. Rhythmic recordings from 3-5 electrodes were smoothed with a 6-hour moving-window average and used to calculate an average peak and trough time for each SCN slice (Figure S1).

*Confocal imaging of GCaMP fluorescence*

On culture day 10, 100% of the culture medium was exchanged and MEA recordings were performed for 3 days (culture days 10-13) to obtain average peak and trough times in action potential activity for each slice. Between culture days 14-21, MEA recordings were repeated for 3 days. During this recording period, MEA probes were temporarily removed for confocal imaging in 30-minute time windows at each peak and trough timepoint to obtain circadian action potential and GCaMP6f $Ca^{2+}$ measurements from the same SCN slice (Figure S1B). Culture

medium exchanges were performed 12 hours prior to conducting simultaneous MEA recording and $Ca^{2+}$ imaging experiments. Fluorescence imaging was performed using a W1 spinning disk confocal on a Ti2 inverted microscope (Nikon) housed at the University of Maryland School of Medicine confocal facility. Probes were temporarily removed from recording headstages during the peak and trough of action potential firing and placed in an environmentally controlled microscope chamber at 37°C with 5% $CO_2$ for confocal imaging to obtain circadian signals in GCaMP6f fluorescence from two full circadian cycles (three troughs and two peaks). GCaMP6f fluorescence was excited for 100 ms with a 488 nm laser and collected with a 515-149 nm emission filter cube. Images (12-bit, 324 nm pixel width and height, no binning) were acquired with a 20× air objective (Nikon) at 10 fps for a duration of 10 seconds and saved as Tiff stack files. LED light intensity was set at 20 to 40% of maximal level, depending on GCaMP6f expression levels.

*Confocal image processing and data analysis*

GCaMP6f images were converted to 8-bit greyscale Tiff files, stacked and aligned in ImageJ (FIJI) (2) using the StackReg plugin (FIJI) (3). Fluorescence intensities from individual neurons within the boundaries of the SCN were measured using oval regions of interest (ROIs) that were manually drawn in ImageJ (FIJI) within the somatic region of individual cells excluding the nucleus, since GCaMP6f expression is cytosolic (4) (Figure S1A). Neurons with apparent baseline fluorescence at least 100× greater than the background fluorescence were used for analysis. For each SCN slice, a minimum of 6 ROIs were drawn in regions devoid of cells and used for background fluorescence measurements. A minimum of 8 cells (one ROI per cell) were analyzed per SCN slice. The average of the mean background fluorescence from 6 ROIs (BGD) was subtracted from the integrated density of the cell ROI fluorescence (Fcell) using the equation: Fcell – (area of ROI × BGD) in Microsoft Excel. Background-subtracted fluorescence intensities for each cell were plotted over time and normalized by dividing the average corrected fluorescence at each timepoint by the maximum value (F/Fmax) of that cell. To calculate the overall F/Fmax for each slice, F/Fmax values from individual cells were averaged within each timepoint, resulting in one F/Fmax value per slice at each timepoint for a total of five values per slice.

**SUPPLEMENTAL FIGURES**

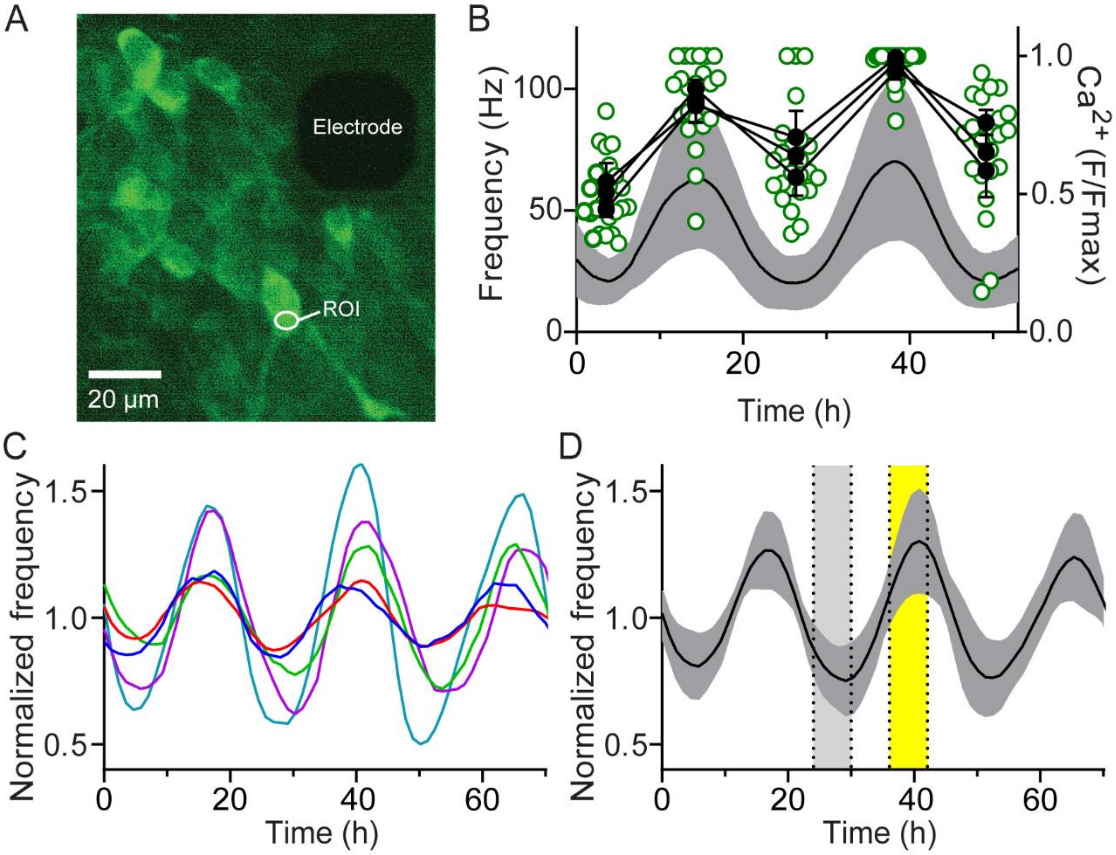


**Figure S1. Establishment of the phase for circadian rhythms in $Ca^{2+}$ and action potential firing.** SCN organotypic slices were transfected with the fluorescent GCaMP6f $Ca^{2+}$ sensor, targeted to neurons using the human synapsin1 (hSyn1) promoter (5). For each slice, action potential firing was recorded for 3 days by multi-electrode array to determine whether slices were rhythmic. The circadian peak and trough were determined from the firing activity rhythm for each slice. Using these as phase reference points, confocal imaging was performed in 30-minute time windows to assess the circadian pattern in intracellular $Ca^{2+}$.

**A)** Confocal image of GCaMP6f $Ca^{2+}$ sensor fluorescence in neurons from an SCN slice cultured on a multielectrode array at 20× magnification with example of a somatic region of interest (ROI) used for analysis in a single neuron (white circle).

**B)** Peak and trough $Ca^{2+}$ measurements superimposed on action potential rhythms, showing $Ca^{2+}$ signals are higher at the peak of firing compared to the trough of firing. This control demonstrates that under these culturing and imaging conditions, the underlying $Ca^{2+}$ signals were rhythmic and diurnal, as prior studies have shown (6-8). Data plots are mean action potential frequency ± SD (black line with grey shading, left axis) recorded from slices on MEAs overlayed with GCaMP6f fluorescence normalized to the maximum (F/Fmax, right axis) measured from the same SCN slices over 3 days. GCaMP6f $Ca^{2+}$ signals from individual neurons (open green circles) and the mean $Ca^{2+}$ signal ± SEM of individual SCN slices (closed black circles) at each timepoint are shown. $Ca^{2+}$ was significantly higher during the peak in action potential firing compared to the trough ($P < 0.0001$, two-way repeated measures ANOVA with Bonferroni *post hoc* test). (N = 3 slices, 8 neurons per slice)

**C)** Circadian rhythms in action potential firing exhibited synchronized phases across the slices in culture, with average peaks and troughs occurring at approximately the same time of the circadian cycle. The firing rate rhythm phases had a standard deviation of 0.7 hours (N = 5 slices). Data plots show action potential firing recorded from SCN slices expressing Venus FLARE-Cameleon on MEAs plotted as frequency smoothed with a 6-h moving window average (normalized to a 24-hr moving window average of raw frequency values) from individual SCN slices (colored lines).

**D)** Since $Ca^{2+}$ rhythms have been reported to peak ~0-5 hours before the peak in action potential firing (27), the indicated 6-hour time window surrounding the average peak and trough times in action potential firing were identified to perform the 30-minute piSPIM imaging experiments detailed in the main text. Data plots are mean action potential frequency ± SD (black line and grey shading) from panel C overlayed with the 6-h time windows at the peak (yellow bar) and trough (grey bar).

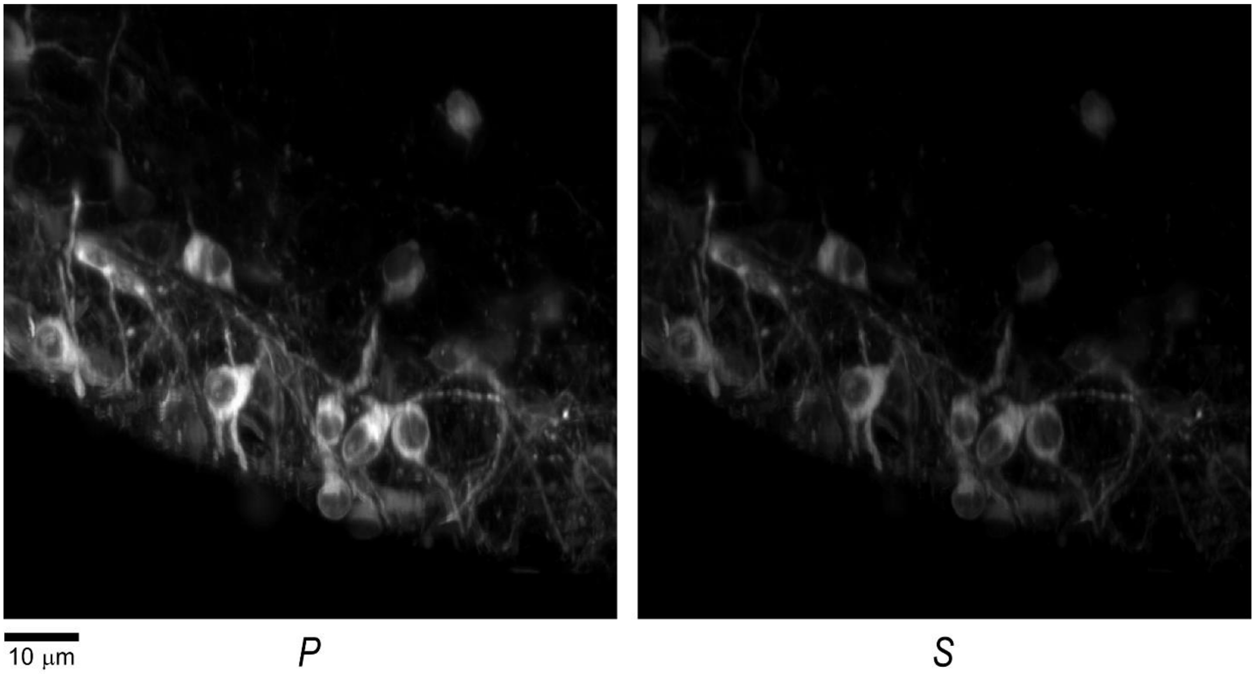


**Figure S2. Three-dimensional projections of piSPIM data.** SCN neurons expressing the Venus-cp172Venus FLARE-Cameleon biosensor. Images represent parallel (*P*) and perpendicular (*S*) z-projections from a representative 20-μm volumetric image stack used for calcium quantification. 3D projections were made using FIJI.

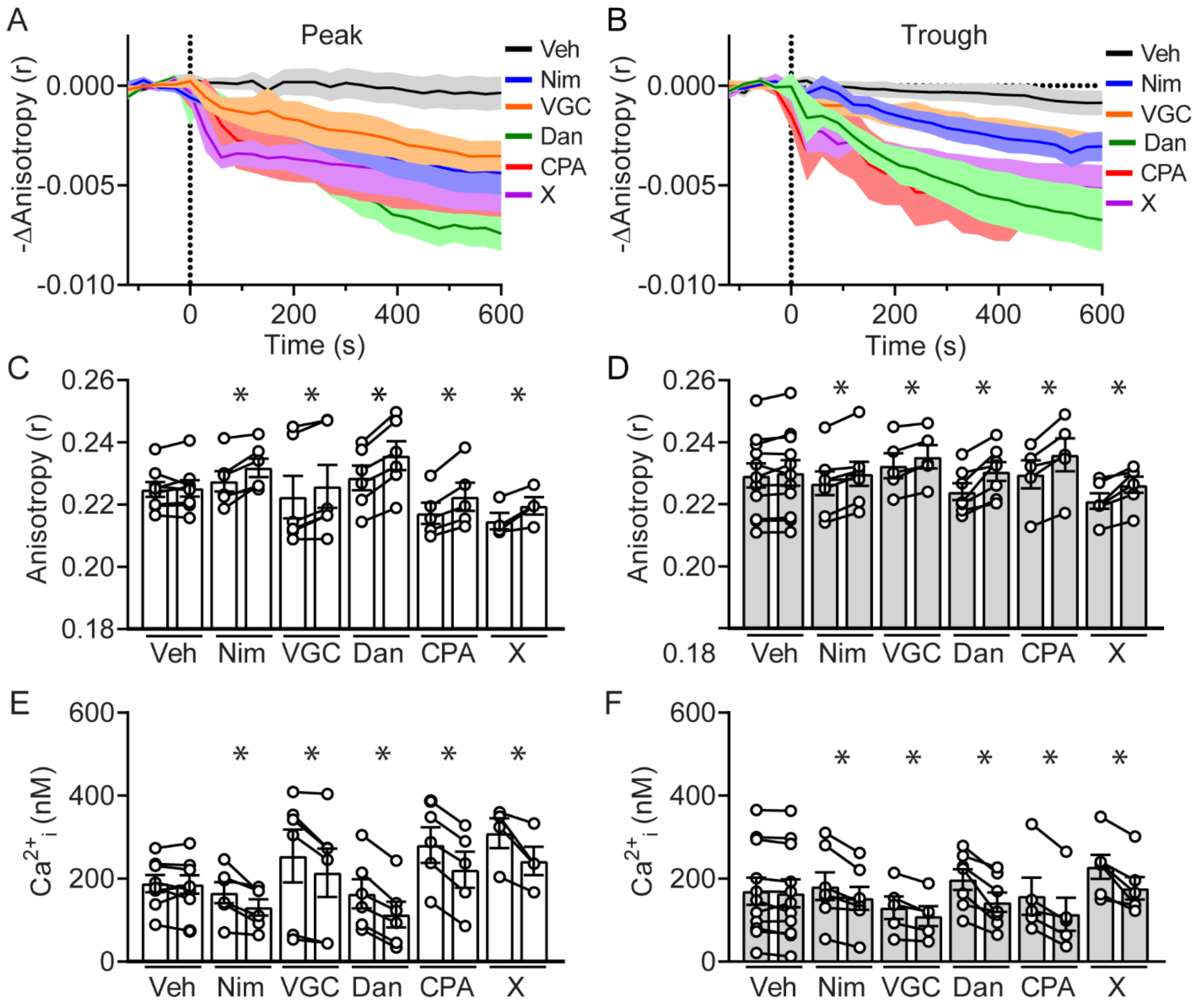


**Figure S3. Effects of $Ca^{2+}$ channel inhibitors on peak and trough anisotropy and $Ca^{2+}$ concentration.** Anisotropy and $Ca^{2+}$ concentrations for data in main Figure 2.

**A, B)** Time course of the negative change in anisotropy (-Δanisotropy = -1 × (anisotropy – average anisotropy from 2 min baseline)) before and after application of $Ca^{2+}$ channel inhibitors at peak (A) and trough (B).

**C, D)** Anisotropy values averaged from baseline recordings and from 9-10 min after application of $Ca^{2+}$ channel inhibitors for each slice at peak (C) or trough (D).

**E, F)** Average $Ca^{2+}$ concentrations at baseline and after application of $Ca^{2+}$ channel inhibitors at peak (D) and trough (F) calculated from anisotropies in C and D. Compared to baseline, anisotropy was increased and $Ca^{2+}$ was decreased by each $Ca^{2+}$ channel inhibitor. *, Significance ($P < 0.05$) tested with paired Student's *t* tests for anisotropy values at peak (Veh, P =0.7; Nim, P = 0.01; VGC, P = 0.007; Dan, P = 0.0005; CPA, P = 0.001; X, P = 0.04) and trough (Veh, P = 0.2; Nim, P = 0.004; VGC, P = 0.01; Dan, P =0.005; CPA, P = 0.008; X, P = 0.009) and for $Ca^{2+}$ concentrations at peak (Veh, P = 0.7; Nim, P = 0.3; VGC, P = 0.1; Dan, P = 0.02; CPA, P = 0.005; X, P = 0.003) and trough (Veh, P = 0.2; Nim, P = 0.01; VGC, P = 0.02; Dan, P =0.004; CPA, P = 0.006; X, P = 0.007). Data are plotted as mean ± SEM. Data points represent paired recordings from individual SCN slices (one imaging region per slice). N= 4-11 slices per drug condition.

## SUPPLEMENTAL REFERENCES

1. McNally, B. A., A. E. Plante, and A. L. Meredith. 2019. Diurnal properties of voltage-gated $Ca^{2+}$ currents in suprachiasmatic nucleus and roles in action potential firing. *J Physiol* 598(9):1775–1790.

2. Schindelin, J., I. Arganda-Carreras, E. Frise, V. Kaynig, M. Longair, T. Pietzsch, S. Preibisch, C. Rueden, S. Saalfeld, B. Schmid, J. Y. Tinevez, D. J. White, V. Hartenstein, K. Eliceiri, P. Tomancak, and A. Cardona. 2012. Fiji: an open-source platform for biological-image analysis. *Nat Methods* 9(7):676-682.

3. Thevenaz, P., U. E. Ruttimann, and M. Unser. 1998. A pyramid approach to subpixel registration based on intensity. *IEEE Trans Image Process* 7(1):27-41.

4. Chen, T. W., T. J. Wardill, Y. Sun, S. R. Pulver, S. L. Renninger, A. Baohan, E. R. Schreiter, R. A. Kerr, M. B. Orger, V. Jayaraman, L. L. Looger, K. Svoboda, and D. S. Kim. 2013. Ultrasensitive fluorescent proteins for imaging neuronal activity. *Nature* 499(7458):295-300.

5. Kugler, S., E. Kilic, and M. Bahr. 2003. Human synapsin 1 gene promoter confers highly neuron-specific long-term transgene expression from an adenoviral vector in the adult rat brain depending on the transduced area. *Gene Ther* 10(4):337-347.

6. Ikeda, M., T. Sugiyama, C. S. Wallace, H. S. Gompf, T. Yoshioka, A. Miyawaki, and C. N. Allen. 2003. Circadian dynamics of cytosolic and nuclear $Ca^{2+}$ in single suprachiasmatic nucleus neurons. *Neuron* 38(2):253-263.

7. Jones, J. R., T. Simon, L. Lones, and E. D. Herzog. 2018. SCN VIP neurons are essential for normal light-mediated resetting of the circadian system. *J Neurosci* 38(37):7986-7995.

8. Enoki, R., D. Ono, S. Kuroda, S. Honma, and K. I. Honma. 2017. Dual origins of the intracellular circadian calcium rhythm in the suprachiasmatic nucleus. *Sci Rep* 7:41733.
